# Forcing axioms and the complexity of non-stationary ideals

**DOI:** 10.1007/s00605-022-01734-w

**Published:** 2022-06-27

**Authors:** Sean Cox, Philipp Lücke

**Affiliations:** 1 Department of Mathematics and Applied Mathematics, Virginia Commonwealth University, 1015 Floyd Avenue, Richmond, Virginia 23284 USA; 2 Facultat de Matemàtiques i Informàtica, Universitat de Barcelona, Gran via de les Corts Catalanes 585, 08007 Barcelona, Spain

**Keywords:** Non-stationary ideals, $\Delta _1$-definability, Martin’s Maximum, Subcomplete Forcing Axiom, stationary reflection, 03E57, 03E35, 03E47

## Abstract

We study the influence of strong forcing axioms on the complexity of the non-stationary ideal on $\omega _2$ and its restrictions to certain cofinalities. Our main result shows that the strengthening $\mathrm{{MM}}^{++}$ of Martin’s Maximum does not decide whether the restriction of the non-stationary ideal on $\omega _2$ to sets of ordinals of countable cofinality is $\Delta _1$-definable by formulas with parameters in $\mathrm{{H}}(\omega _3)$. The techniques developed in the proof of this result also allow us to prove analogous results for the full non-stationary ideal on $\omega _2$ and strong forcing axioms that are compatible with $\mathrm{{CH}}$. Finally, we answer a question of S. Friedman, Wu and Zdomskyy by showing that the $\Delta _1$-definability of the non-stationary ideal on $\omega _2$ is compatible with arbitrary large values of the continuum function at $\omega _2$.

## Introduction

The fact that closed unbounded subsets generate a proper normal filter, the *club filter* on $\kappa$

$$\begin{aligned} {{Club}}_{\kappa } ~ = ~ \{{A\subseteq \kappa }~\vert ~{\exists C\subseteq A\, closed\, and\, unbounded\, in\, \kappa }\}, \end{aligned}$$

is one of the most important combinatorial properties of uncountable regular cardinals $\kappa$. The study of the structural properties of these filters and their dual ideals, the *non-stationary ideal* on $\kappa$

$$\begin{aligned} {{NS}}_{\kappa } ~ = ~ \{{A\subseteq \kappa }~\vert ~{{\exists C { closed\, and\, unbounded\, in }\, \kappa {\, with \,} A\cap C=\emptyset }}\} \end{aligned}$$

plays a central role in modern set theory.

In [23] and [24], Mekler, Shelah and Väänänen initiated the study of the *complexity* of club filters and non-stationary ideals, leading to various results establishing interesting connections between the complexity of these objects and their structural properties. Given an uncountable regular cardinal $\kappa$, it is easy to see that both ${{Club}}_{\kappa }$ and ${{NS}}_{\kappa }$ are definable by a $\Sigma _1$-formula with parameter $\kappa$, i.e. there exist $\Sigma _1$-formulas $\varphi _0(v_0,v_1)$ and $\varphi _1(v_0,v_1)$ such that ${{Club}}_{\kappa }=\{{A}~\vert ~{\varphi _0(A,\kappa )}\}$ and ${{NS}}_{\kappa }=\{{A}~\vert ~{\varphi _1(A,\kappa )}\}$. The results of [24] show that under $\mathrm{{CH}}$, the $\varvec{\Delta }_1(\mathrm{{H}}(\omega _2))$-definability of ${{NS}}_{\omega _1}$ (i.e. the assumption that ${{NS}}_{\omega _1}=\{{A}~\vert ~{\psi _1(A,z)}\}$ holds for some $\Pi _1$-formula $\psi (v_0,v_1)$ and some $z\in \mathrm{{H}}(\omega _2)$) is equivalent to several interesting combinatorial and model-theoretic assumptions about objects of size $\omega _1$. In particular, it is shown that this definability assumption is equivalent to the existence of a so-called *canary tree*, a tree of height and cardinality $\omega _1$ without cofinal branches that has specific properties with respect to the ordering of such trees under order-preserving embeddings. Since the results of [23] show that the existence of a canary tree is independent of $\mathrm{{ZFC}}+\mathrm{{CH}}$, it follows that this theory is not able to determine the exact complexity of ${{NS}}_{\omega _1}$.

The above results were later generalized to higher cardinals. If *S* is a stationary subset of an uncountable regular cardinal $\kappa$, then we let ${{NS}}_{}\restriction S={{NS}}_{\delta }\cap {\wp }({S})$ denote the *restriction* of the non-stationary ideal on $\delta$ to *S*. Given infinite regular cardinals $\lambda <\kappa$, we set $S^\kappa _\lambda =\{{\alpha <\kappa }~\vert ~{{\mathrm{{cof}}(\alpha )}=\lambda }\}$. In addition, if $m<n<\omega$, then we write $S^n_m$ instead of $S^{\omega _n}_{\omega _m}$. Results of Hyttinen and Rautila in [13] showed that if $\kappa$ is an infinite regular cardinal in a model of the $\mathrm{{GCH}}$, then, in a cofinality-preserving forcing extension, the set ${{NS}}_{}\restriction S^{\kappa ^+}_\kappa$ is $\varvec{\Delta }_1(\mathrm{{H}}(\kappa ^{++}))$-definable. Furthermore, in [10], S. Friedman, Wu and Zdomskyy showed that for every successor cardinal in Gödel’s constructible universe $\mathrm{{L}}$, there is a cardinality-preserving forcing extension of $\mathrm{{L}}$ in which ${{NS}}_{\kappa }$ is $\varvec{\Delta }_1(\mathrm{{H}}(\kappa ^+))$-definable. These results can be easily used to show that the complexity of the non-stationary ideal and its restriction is not determined by $\mathrm{{ZFC}}$ (see Lemma 1.1 and the subsequent discussion below). Finally, recent work also unveiled several interesting consequences of the $\varvec{\Delta }_1(\mathrm{{H}}(\kappa ^+))$-definability of restriction of ${{NS}}_{\kappa }$ at higher cardinals $\kappa$. In particular, this set-theoretic assumption was shown to be closely connected to model-theoretic questions dealing with Shelah’s *Classification Theory* and the complexity of certain mathematical theories (see, for example, [8,  Theorem 64]).

The above results strongly motivate the question whether canonical extensions of $\mathrm{{ZFC}}$ decide more about the complexity of non-stationary ideals, and this question turns out to be closely connected to important recent developments in set theory. In [8], S. Friedman, Hyttinnen and Kulikov showed that, in the constructible universe $\mathrm{{L}}$, the sets of the form ${{NS}}_{}\restriction S$ for some stationary subset *S* of an uncountable regular cardinal $\kappa$ are not $\varvec{\Delta }_1(\mathrm{{H}}(\kappa ^+))$-definable. Using the notion of *local club condensation* (see [7]), it is possible to extend this conclusion to larger canonical inner models. In another direction, S. Friedman and Wu observed in [9] that strong saturation properties of the non-stationary ideal on $\omega _1$, i.e. the assumption that the poset ${\wp }({\omega _1})/{{NS}}_{\omega _1}$ has a dense subset of cardinality $\omega _1$, imply the $\varvec{\Delta }_1(\mathrm{{H}}(\omega _2))$-definability of ${{NS}}_{\omega _1}$. Results of Woodin in [26,  Chapter 6] show that ${{NS}}_{\omega _1}$ possesses these properties in certain forcing extensions of determinacy models. Finally, Schindler and his collaborators recently studied the question whether forcing axioms determine the complexity of ${{NS}}_{\omega _1}$. In [19], Larson, Schindler and Wu showed that *Woodin’s Axiom* $(*)$ (see [26,  Definition 5.1]) implies that ${{NS}}_{\omega _1}$ is not $\varvec{\Delta }_1(\mathrm{{H}}(\omega _2))$-definable. In combination with recent results of Asperó and Schindler in [1], this shows that $\mathrm{{MM}}^{++}$, a natural strengthening of *Martin’s Maximum*, implies that ${{NS}}_{\omega _1}$ is not $\varvec{\Delta }_1(\mathrm{{H}}(\omega _2))$-definable.

The work presented in this paper is motivated by the question whether strong forcing axioms determine the complexity of the non-stationary ideal on $\omega _2$ and its restrictions. The following result from [21] shows that all extensions of $\mathrm{{ZFC}}$ that are preserved by forcing with ${<}\omega _2$-directed posets are compatible with the assumption that for every stationary subset *S* of $\omega _2$, the set ${{NS}}_{}\restriction S$ is not $\varvec{\Delta }_1(\mathrm{{H}}(\omega _3))$-definable. In particular, the results of [4, 17, 18] show that this statement is compatible with all standard forcing axioms, like $\mathrm{{MM}}^{++}$. The lemma follows directly from a combination of [21,  Theorem 2.1], showing that no $\varvec{\Delta }^1_1$-definable set (see [20,  Definition 1.2]) separates ${{Club}}_{\kappa }$ from ${{NS}}_{\kappa }$ in the given model of set theory, and [20,  Lemma 2.4], showing that $\varvec{\Delta }^1_1$-definability coincides with $\varvec{\Delta }_1(\mathrm{{H}}(\kappa ^+))$-definability at all uncountable regular cardinals $\kappa$.

### Lemma 1.1

Let $\kappa$ be an uncountable cardinal with $\kappa ^{{<}\kappa }=\kappa$ and let *G* be $\mathrm{{Add}}({\kappa },{\kappa ^+})$-generic over $\mathrm{{V}}$. In $\mathrm{{V}}[G]$, no $\varvec{\Delta }_1(\mathrm{{H}}(\kappa ^+))$-definable subset of ${\wp }({\kappa })$ separates ${{Club}}_{\kappa }$ from ${{NS}}_{\kappa }$, i.e. no set *X* definable in this way satisfies ${{Club}}_{\kappa }\subseteq X\subseteq {\wp }({\kappa })\setminus {{NS}}_{\kappa }$.

Note that, if *S* is a stationary subset of an uncountable regular cardinal $\kappa$, then ${{NS}}_{}\restriction S$ separates ${{Club}}_{\kappa }$ from ${{NS}}_{\kappa }$. This shows that, in $\mathrm{{Add}}({\kappa },{\kappa ^+})$-generic extensions, sets of the form ${{NS}}_{}\restriction S$ for stationary subsets *S* are not $\varvec{\Delta }_1(\mathrm{{H}}(\kappa ^+))$-definable.

In contrast, we will prove the following theorem that shows that strong forcing axioms like $\mathrm{{MM}}^{++}$ are also compatible with the existence of a $\varvec{\Delta }_1(\mathrm{{H}}(\omega _3))$-definable set that separates the club filter on $\omega _2$ from the corresponding non-stationary ideal. The proof of this result is based on a detailed analysis of the preservation properties of a variation of a forcing iteration constructed by Hyttinen and Rautila in the consistency proofs of [13]. Our construction will also allow us to produce such models with arbitrary large $2^{\omega _2}$. See Sect. 2 for the meaning of the “$+\mu$” versions of forcing axioms.^1^

### Theorem 1.2

Let $\mathrm{{FA}}$ denote any one of the following forcing axioms:

- $\mathrm{{MM}}^{{+}\mu }$, where $\mu$ is a cardinal and $0 \le \mu \le \omega _1$; or
- $\mathrm{{PFA}}^{{+}\mu }$, where $\mu$ is a cardinal and $1 \le \mu \le \omega _1$.

Assume that $\mathrm{{FA}}$ holds, and let $\theta$ be a cardinal with $\theta ^{\omega _2}=\theta$. Then there exists a ${<}\omega _2$-directed closed, cardinal-preserving poset ${\mathbb {P}}$ with the property that whenever *G* is ${\mathbb {P}}$-generic over $\mathrm{{V}}$, then, in $\mathrm{{V}}[G]$, the axiom $\mathrm{{FA}}$ still holds, $2^{\omega _2}=\theta$ and the set ${{NS}}_{}\restriction S^2_0$ is $\varvec{\Delta }_1(\mathrm{{H}}(\omega _3))$-definable.

We will also apply the techniques developed in the proof of the above result to forcing axioms that are compatible with the continuum hypothesis, focusing on the axiom $\mathrm{{FA}}^{{+}}(\sigma \text {-closed})$ and the *subcomplete forcing axiom* $\mathrm {SCFA}$ introduced by Jensen in [16]. Note that both axioms are preserved by ${<}\omega _2$-directed closed forcings (see [4] and [18]) and hence Lemma 1.1 above already shows that they are compatible with the assumption that no $\varvec{\Delta }_1(\mathrm{{H}}(\omega _3))$-definable set separates ${{Club}}_{\omega _2}$ from ${{NS}}_{\omega _2}$.

### Theorem 1.3

Let $\mathrm{{FA}}$ denote either the axiom $\mathrm {SCFA}$ or the axiom $\mathrm{{FA}}^{{+}}(\sigma \text {-closed})$. Assume that $2^\omega =\omega _1$, $2^{\omega _1}=\omega _2$ and $\mathrm{{FA}}$ holds. Let $\theta$ be a cardinal satisfying $\theta ^{\omega _2}=\theta$. Then there exists a ${<}\omega _2$-directed closed, cardinal-preserving poset ${\mathbb {P}}$ with the property that whenever *G* is ${\mathbb {P}}$-generic over $\mathrm{{V}}$, then, in $\mathrm{{V}}[G]$, the axiom $\mathrm{{FA}}$ still holds, $2^{\omega _2}=\theta$ and the set ${{NS}}_{\omega _2}$ is $\varvec{\Delta }_1(\mathrm{{H}}(\omega _3))$-definable.

This theorem also provides an affirmative answer to [10,  Problem 3.3] posed by S. Friedman, Wu and Zdomskyy, by showing that the $\varvec{\Delta }_1$-definability of ${{NS}}_{\omega _2}$ is compatible with $2^{\omega _2}\ge \omega _5$.

## Preliminaries

This section covers well-known results, mostly related to the notions of internal approachability and Shelah’s Approachability Ideal.

First we recall the “plus” versions of forcing axioms, which were first introduced by Baumgartner [2] (though the prevailing notation has changed somewhat since then). If $\Gamma$ is a class of posets and $\mu$ is a cardinal, $\text {FA}^{+\mu }\big (\Gamma \big )$ states that for every poset ${\mathbb {P}}\in \Gamma$, for every collection $\mathcal {D}$ of size $\omega _1$ of dense subsets of ${\mathbb {P}}$ and for every sequence $\langle {\sigma _\xi }~\vert ~{\xi <\mu }\rangle$ of ${\mathbb {P}}$-names for stationary subsets of $\omega _1$, there is a $\mathcal {D}$-generic filter *g* on ${\mathbb {P}}$ with the property that the set $\{{\alpha <\omega _1}~\vert ~{\exists p\in g ~ p\Vdash _{\mathbb {P}}{\text {``}{\check{\alpha }\in \sigma _\xi }\text {''}}}\}$ is stationary in $\omega _1$ for every $\xi <\mu$. If $\Gamma$ is the class of posets that preserve stationary subsets of $\omega _1$, we write $\mathrm{{MM}}^{+\mu }$ instead of $\text {FA}^{+\mu }(\Gamma )$; and, as mentioned earlier, $\mathrm{{MM}}^{++}$ refers to $\mathrm{{MM}}^{+\omega _1}$, *not* to $\mathrm{{MM}}^{+2}$. Similar comments apply to the class of proper posets and PFA.

### Definition 2.1

Let ${\mathbb {P}}$ be a poset and let $W \prec (\mathrm{{H}}(\theta ),\in ,{\mathbb {P}})$ for some sufficiently large regular cardinal $\theta$.

1. A condition $p \in {\mathbb {P}}$ is a $\varvec{(W,{\mathbb {P}})}$**-master condition** if
  $$\begin{aligned} W[G]\cap \mathrm{{V}}~ = ~ W \end{aligned}$$
  holds whenever *G* is ${\mathbb {P}}$-generic over $\mathrm{{V}}$ with $p\in G$.
2. A set *g* is $\varvec{(W,{\mathbb {P}})}$**-generic** if $g \subseteq {\mathbb {P}}\cap W$, *g* is a filter on ${\mathbb {P}}\cap W$, and $D\cap g \ne \emptyset$ for every $D \in W$ that is a dense subset of ${\mathbb {P}}$.^2^
3. A condition $p \in \mathbb {P}$ is a $\varvec{(W,{\mathbb {P}})}$**-total master condition** if the set
  $$\begin{aligned} \{{r \in {\mathbb {P}}\cap W}~\vert ~{p\le _{\mathbb {P}}r}\} \end{aligned}$$
  is a $(W,{\mathbb {P}})$-generic filter.

The following result is well-known:

### Lemma 2.2

Let ${\mathbb {P}}$ be a poset, let $W \prec (\mathrm{{H}}(\theta ),\in ,{\mathbb {P}})$, let $\mu$ be an ordinal with $\mu \subseteq W$, and let $\dot{f} \in W$ be a ${\mathbb {P}}$-name for a function from $\mu$ to the ground model $\mathrm{{V}}$. If *p* is a $(W,{\mathbb {P}})$-total master condition and *G* is ${\mathbb {P}}$-generic over $\mathrm{{V}}$ with $p\in G$, then $\dot{f}^G\in \mathrm{{V}}$.

### Proof

Fix a $(W,{\mathbb {P}})$-total master condition *p* and a filter *G* on ${\mathbb {P}}$ that is generic over $\mathrm{{V}}$ and contains the condition *p*. Let $g=\{{r \in {\mathbb {P}}\cap W}~\vert ~{p\le _{\mathbb {P}}r}\}$ denote the $(W,{\mathbb {P}})$-generic filter induced by *p*. Then $G \cap W$ is a $(W,{\mathbb {P}})$-generic filter extending *g* and therefore standard arguments show that $G \cap W=g$. By elementarity, there is a sequence $\langle {A_\xi }~\vert ~{\xi <\mu }\rangle \in W$ of maximal antichains in ${\mathbb {P}}$ with the property that for every $\xi <\mu$, each condition in $A_\xi$ decides the value of $\dot{f}$ at $\xi$. Since $\mu \subseteq W$, this shows that for all $\xi <\mu$, the unique condition in $A_\xi \cap g$ decides the value of $\dot{f}$ at $\xi$. But the sequence $\langle {A_\xi }~\vert ~{\xi <\mu }\rangle$ and the filter *g* are both elements of $\mathrm{{V}}$ and hence the function $\dot{f}^G$ is also in the ground model. $\square$

We state a definition that will be used extensively in the following arguments:

### Definition 2.3

Given an infinite regular cardinal $\kappa$, we let $\text {IA}_\kappa$ denote the class of all sets *W* with the property that there exists a sequence $\vec {N} = \langle {N_\alpha }~\vert ~{\alpha < \kappa }\rangle$ that satisfies the following statements:

1. The sequence $\vec {N}$ is $\subseteq$-increasing and $\subseteq$-continuous.
2. $$W = \bigcup \{{N_\alpha }~\vert ~{\alpha < \kappa }\}$$.
3. $$\vert {N_\alpha }\vert < \kappa $$ for all $$\alpha <\kappa $$.
4. Every proper initial segment of $\vec {N}$ is an element of *W*.

### Remark 2.4

If $\vec {N}$ witnesses that *W* is an element of $\text {IA}_\kappa$ and $W \prec \mathrm{{H}}(\theta )$ for some $\theta > \kappa$, then $\kappa \subseteq W$. This is because we have $\vec {N} \restriction \alpha \in W$ for every $\alpha < \kappa$, and the domain of $\vec {N} \restriction \alpha$, namely $\alpha$, is definable from the parameter $\vec {N} \restriction \alpha$.

In what follows, if $\tau$ is a regular uncountable cardinal, ${\wp }_{\tau }(H)$ refers to the set of all $W \subseteq H$ with $\vert {W}\vert <\tau$, and ${\wp }^*_{\tau }(H)$ denotes the set

$$\begin{aligned} \{{W \in {\wp }_{\tau }(H)}~\vert ~{W \cap \tau \in \tau }\}. \end{aligned}$$

The set ${\wp }^*_{\tau }(\mathrm{{H}}(\theta ))$ contains a club in the sense of Jech (see [14]), but not necessarily in the sense of Shelah (see [6]).

### Remark 2.5

In the above situation, if $W \in {\wp }^*_{\tau }(\mathrm{{H}}(\theta ))$, $W \prec \mathrm{{H}}(\theta )$, and $x \in W$ with $\vert {x}\vert < \tau$, then $x \subseteq W$.^3^

### Lemma 2.6

If $\kappa$ is a regular and uncountable cardinal, then $\text {IA}_\kappa$ is stationary in ${\wp }_{\kappa ^+}(\mathrm{{H}}(\theta ))$ for all sufficiently large regular $\theta$.

### Proof

Given a first-order structure $\mathfrak {A} = (\mathrm{{H}}(\theta ), \in , \kappa , \ldots )$ in a countable language, recursively construct a $\subseteq$-continuous and $\subseteq$-increasing sequence $\vec {N} = \langle {N_\alpha }~\vert ~{\alpha < \kappa }\rangle$ of elementary substructures of $\mathfrak {A}$ of cardinality less than $\kappa$ such that $\vec {N} \restriction \alpha \in N_{\alpha +1}$ for all $\alpha < \kappa$. Then $\vec {N}$ witnesses that its union is contained in $\text {IA}_\kappa$. $\square$

### Lemma 2.7

Let ${\mathbb {P}}$ be a poset, let $\kappa <\theta$ be infinite regular cardinals with ${\mathbb {P}}\in \mathrm{{H}}(\theta )$, let $\lhd$ be a well-ordering of $\mathrm{{H}}(\theta )$, let $W \prec (\mathrm{{H}}(\theta ),\in ,{\mathbb {P}},\lhd )$ with $W \in \text {IA}_\kappa$, and let $p \in {\mathbb {P}}\cap W$.

1. If ${\mathbb {P}}$ is ${<}\kappa$-closed, then there exists a $(W,{\mathbb {P}})$-generic filter that contains *p*.
2. If ${\mathbb {P}}$ is ${<}\kappa ^+$-closed, then there exists a $(W,{\mathbb {P}})$-total master condition below *p*.

### Proof

Let $\vec {N} = \langle {N_\alpha }~\vert ~{\alpha < \kappa }\rangle$ witness that *W* is an element of $\text {IA}_\kappa$.

(1) Assuming that ${\mathbb {P}}$ is ${<}\kappa$-closed. Using the closure of ${\mathbb {P}}$ and the fact that each $N_\alpha$ has cardinality less than $\kappa$, we can recursively construct a descending sequence $\vec {p}=\langle {p_\alpha }~\vert ~{\alpha < \kappa }\rangle$ of conditions below *p* in ${\mathbb {P}}$ such that the following statements hold for all $\alpha < \kappa$:

- The condition $p_{\alpha +1}$ is the $\lhd$-least element of ${\mathbb {P}}$ below $p_\alpha$ that is an element of every open dense set that belongs to $N_\alpha$.^4^
- If $\alpha$ is a limit ordinal, then $p_\alpha$ is the $\lhd$-least lower bound of the sequence $\langle {p_\ell }~\vert ~{\ell < \alpha }\rangle$.

Then every proper initial segment of $\vec {p}$ is definable from a proper initial segment of $\vec {N}$, and hence every proper initial segment of $\vec {p}$ is in *W*. In particular, we know that $p_{\alpha +1} \in W$ for all $\alpha < \kappa$. It follows that the filter in ${\mathbb {P}}$ generated by the subset $\{{p_\alpha }~\vert ~{\alpha < \kappa }\}$ is $(W,{\mathbb {P}})$-generic.

(2) Now, assume that ${\mathbb {P}}$ is ${<}\kappa ^+$-closed and repeat the above construction of the sequence $\vec {p}$. Then $\vec {p}$ has a lower bound in ${\mathbb {P}}$, and this lower bound is clearly a $(W,{\mathbb {P}})$-total master condition. $\square$

Next we discuss one variant of proper forcing.

### Definition 2.8

Let $\kappa$ be an infinite regular cardinal.

1. A poset ${\mathbb {P}}$ is $\varvec{IA}_{\varvec{\kappa }}$**-proper** if for all sufficiently large regular cardinals $\theta$, all $W \prec (\mathrm{{H}}(\theta ),\in ,{\mathbb {P}})$ with $W \in \text {IA}_\kappa$ and all $p \in {\mathbb {P}}\cap W$, there is a $(W,\mathbb {P})$-master condition below *p*.
2. A poset ${\mathbb {P}}$ is $\varvec{IA}_{\varvec{\kappa }}$**-totally proper** if for all sufficiently large regular cardinals $\theta$, all $W \prec (\mathrm{{H}}(\theta ),\in ,{\mathbb {P}})$ with $W \in \text {IA}_\kappa$ and all $p \in {\mathbb {P}}\cap W$, there is a $(W,\mathbb {P})$-total master condition below *p*.

It is well-known that $\text {IA}_\kappa$-proper posets preserve all stationary subsets of $S^{\kappa ^+}_\kappa$ that lie in the approachability ideal $I[\kappa ^+]$ defined below. Since we could not find a reference for exactly what is needed in our arguments, we sketch the proof below. Note that it is possible for $\text {IA}_\kappa$-proper (even $\text {IA}_\kappa$-totally proper) posets to destroy the stationarity of some subsets of $S^{\kappa ^+}_\kappa$ (see [5]). So $\text {IA}_\kappa$-total properness is, in general, strictly weaker than $\kappa ^+$-Jensen completeness (defined in the next section), because ${<}\kappa ^+$-closed forcings preserve all stationary subsets of $\kappa ^+$.

### Definition 2.9

(Shelah) Let $\kappa$ be an infinite regular cardinal.

1. Given a sequence $\vec {z} = \langle {z_\alpha }~\vert ~{\alpha < \kappa ^+}\rangle$ a sequence of elements of $[\kappa ^+]^{{<}\kappa }$, an ordinal $\gamma < \kappa ^+$ is called **approachable with respect to** $\varvec{\vec {z}}$ if there exists a sequence
  $$\begin{aligned} \vec {\alpha } ~ = ~ \langle {\alpha _\xi }~\vert ~{\xi < {\mathrm{{cof}}(\gamma )}}\rangle \end{aligned}$$
  cofinal in $\gamma$ such that every proper initial segment of $\vec {\alpha }$ is equal to $z_\alpha$ for some $\alpha < \gamma$.
2. The **Approachability ideal** $\varvec{I[\kappa ^+]}$ on $\kappa ^+$ is the (possibly non-proper) *normal* ideal generated by sets of the form
  $$\begin{aligned} A_{\vec {z}} ~ = ~ \{{\gamma < \kappa ^+}~\vert ~{{\gamma \, is\, approachable\, with\, respect\, to\, \vec {z} }}\} \end{aligned}$$
  for some sequence $\vec {z} \in {}^{\kappa ^+}([\kappa ^+]^{{<}\kappa })$.

Note that a subset *X* of $\kappa ^+$ is an element of $I[\kappa ^+]$ if and only if there exists some club $D \subseteq \kappa ^+$ and some sequence $\vec {z} \in {}^{\kappa ^+}([\kappa ^+]^{{<}\kappa })$ such that every $\gamma \in D \cap X$ is approachable with respect to $\vec {z}$. In the following, we will make use of several facts about $I[\kappa ^+]$. Throughout this section, $\kappa$ denotes a regular cardinal.

### Lemma 2.10

([5]) Suppose $\kappa ^{{<}\kappa } \le \kappa ^+$, and let $\langle {z_ \alpha }~\vert ~{\alpha < \kappa ^+}\rangle$ be an enumeration of $[\kappa ^+]^{{<}\kappa }$.^5^ Define

$$\begin{aligned} M_{\vec {z}} ~ = ~ \{{\gamma \in S^{\kappa ^+}_\kappa }~\vert ~{{\gamma \, is\, approachable\, with\, respect\, to\, \vec {z}}}\}. \end{aligned}$$

Then the following statements hold:

1. $M_{\vec {z}}$ is a stationary subset of $S^{\kappa ^+}_\kappa$.
2. $$M_{\vec {z}} \in I[\kappa ^+]$$.
3. $M_{\vec {z}}$ is a *maximum element of* $I[\kappa ^+]\cap {\wp }({S^{\kappa ^+}_\kappa })$ *mod NS*, i.e. whenever *S* is a stationary subset of $S^{\kappa ^+}_\kappa$ such that $S \in I[\kappa ^+]$, then $S \setminus M_{\vec {z}}$ is non-stationary.
4. If $\kappa ^{{<}\kappa } = \kappa$, then $S^{\kappa ^+}_\kappa \setminus M_{\vec {z}}$ is non-stationary. In particular, $\kappa ^{{<}\kappa } = \kappa$ implies that $S^{\kappa ^+}_\kappa \in I[\kappa ^+]$.

### Proof

(1) Fix a sufficiently large regular cardinal $\theta$ and a well-ordering $\lhd$ of $\mathrm{{H}}(\theta )$. Fix $W \in \text {IA}_\kappa$ with $W \prec (\mathrm{{H}}(\theta ),\in ,\lhd ,\vec {z})$ and let $\langle {N_\alpha }~\vert ~{\alpha < \kappa }\rangle$ be a sequence witnessing that $W \in \text {IA}_\kappa$. Given $\alpha <\kappa$, set $\gamma _\alpha =\sup (N_\alpha \cap \kappa ^+)<\kappa ^+$. Then $\vec {\gamma }=\langle {\gamma _\alpha }~\vert ~{\alpha <\kappa }\rangle$ enumerates a cofinal subset of $W \cap \kappa ^+$ of order-type $\kappa$ and every proper initial segment of this sequence is an element of *W*.

Moreover, each proper initial segment of $\vec {\gamma }$ is an element of $[\kappa ^+]^{{<}\kappa }$, and hence an element of

$$\begin{aligned} W \cap \{{ z_\alpha }~\vert ~{\alpha< \kappa ^+}\} ~ = ~ \{{z_\alpha }~\vert ~{\alpha < W \cap \kappa ^+}\}. \end{aligned}$$

This shows that $W \cap \kappa ^+$ is approachable with respect to $\vec {z}$. Since Lemma 2.6 shows that there are stationarily-many $W \in \text {IA}_\kappa$ with $W \prec (\mathrm{{H}}(\theta ),\in ,\lhd ,\vec {z})$, these computations allow us to conclude that $M_{\vec {z}}$ is a stationary subset of $S^{\kappa ^+}_\kappa$.

(2) Since $M_{\vec {z}}\subseteq A_{\vec {z}} \in I[\kappa ^+]$, the statement $M_{\vec {z}} \in I[\kappa ^+]$ holds trivially.

(3) Now, suppose that $S \in I[\kappa ^+]$ is a stationary subset of $S^{\kappa ^+}_\kappa$. By earlier remarks, there is a sequence $\vec {u} = \langle {u_\alpha }~\vert ~{\alpha < \kappa ^+}\rangle$ of elements of $[\kappa ^+]^{{<}\kappa }$ and club subset *D* of $\kappa ^+$ with the property that every $\gamma \in D \cap S$ is approachable with respect to $\vec {u}$. Define

$$\begin{aligned} E ~ = ~ \{{\gamma \in S}~\vert ~{\text {Hull}^{(\mathrm{{H}}(\theta ),\in ,\vec {u},\vec {z},D)}(\gamma ) \cap \kappa ^+ = \gamma }\}. \end{aligned}$$

Then $S\setminus E$ is non-stationary. Fix $\gamma \in E$ and set $M(\gamma )= \text {Hull}^{(\mathrm{{H}}(\theta ),\in ,\vec {u},\vec {z},D)}(\gamma )$. Since $\gamma \in S\in I[\kappa ^+]$, there is a cofinal sequence $\vec {\beta }=\langle {\beta _\ell }~\vert ~{\ell < \kappa }\rangle$ in $\gamma$ such that every proper initial segment of $\vec {\beta }$ appears in $\vec {u}\restriction \gamma$. But since $\vec {z}$ enumerates all of $[\kappa ^+]^{{<}\kappa }$, the fact that $\vec {u}, \vec {z} \in M(\gamma ) \prec (\mathrm{{H}}(\theta ),\in )$ implies that for every $\alpha < \gamma$ there is a $k(\alpha ) < \gamma$ with $u_\alpha = z_{k(\alpha )}$, i.e.

$$\begin{aligned} \{{u_\alpha }~\vert ~{\alpha< \gamma }\} ~ \subseteq ~ \{{z_\alpha }~\vert ~{\alpha < \gamma }\}. \end{aligned}$$

In particular, every proper initial segment of $\vec {\beta }$ appears in $\vec {z}$ before $\gamma$ and therefore $\gamma$ is approachable with respect to $\vec {z}$. These computations show that $S\setminus M_{\vec {z}}\subseteq S\setminus E$ is non-stationary in $\kappa ^+$.

(4) Now, assume that $\kappa ^{{<}\kappa } = \kappa$. Then $\vert {\eta ^{{<}\kappa }}\vert = \kappa$ for every $\eta < \kappa ^+$ and hence there is a function ${f}:{\kappa ^+}\longrightarrow {\kappa ^+}$ with the property that for all $\eta <\kappa ^+$, every element of $[\eta ]^{{<}\kappa }$ is enumerated by $\vec {z} \restriction f(\eta )$. Let *D* denote the club of all $\kappa<\gamma < \kappa ^+$ such that

$$\begin{aligned} \text {Hull}^{(\mathrm{{H}}(\theta ),\in ,\vec {z},f)}(\gamma ) ~ \cap ~ \kappa ^+ ~ = ~ \gamma . \end{aligned}$$

Pick $\gamma \in D \cap S^{\kappa ^+}_\kappa$, and set $W(\gamma )= \text {Hull}^{(\mathrm{{H}}(\theta ),\in ,\vec {z},f)}(\gamma )$. Fix a cofinal sequence $\vec {\alpha }$ in $\gamma$ of order-type $\kappa$ in $\gamma$, and some $\xi < \kappa$. Since ${\mathrm{{cof}}(\gamma )} = \kappa$, there is $\eta < \gamma$ with $\alpha _\ell <\eta$ for all $\ell <\xi$ and $\vec {\alpha } \restriction \xi = z_\zeta$ for some $\zeta < f(\eta )$. Moreover, since $\eta \in W(\gamma )$ and $\vert {f(\eta )}\vert \le \kappa \subseteq W(\gamma )$, elementarity implies that $f(\eta ) \in W(\gamma )$ and $f(\eta )\subseteq W(\gamma )$. Since $\vec {z}$ and $\zeta$ are both elements of $W(\gamma )$, we can conclude that $z_\zeta \in W(\gamma )$. Hence $\gamma$ is approachable with respect to $\vec {z}$. $\square$

The next few lemmas address stationary set preservation when GCH may fail to hold.

### Lemma 2.11

The class $\text {IA}_{\kappa }$ is projective stationary over

$$\begin{aligned} \mathcal {S}~ = ~\{{T \subseteq S^{\kappa ^+}_\kappa }~\vert ~{{T\, is \,stationary\, and\, T \in I[\kappa ^+]}}\}, \end{aligned}$$

i.e. if $T \in \mathcal {S}$, then for every sufficiently large regular cardinal $\theta$ and every function ${F}:{[\mathrm{{H}}(\theta )]^{{<}\omega }}\longrightarrow {\mathrm{{H}}(\theta )}$, there exists $W \in \text {IA}_\kappa$ such that $W \cap \kappa ^+ \in T$ and *W* is closed under *F*.

### Proof

Fix $T\in \mathcal {S}$. Then there is a club *D* in $\kappa ^+$ and a sequence $\vec {z}\in {}^{\kappa ^+}[\kappa ^+]^{{<}\kappa }$ such that every element of $D \cap T$ is approachable with respect to $\vec {z}$.

Fix a regular $\vartheta$ with $F \in \mathrm{{H}}(\vartheta )$, let $\lhd$ be a well-ordering of $\mathrm{{H}}(\vartheta )$ and set

$$\begin{aligned} \mathfrak {A} ~ = ~ (\mathrm{{H}}(\vartheta ),\in ,\lhd ,\vec {z},D,F, T). \end{aligned}$$

Pick $\gamma \in D \cap T$ and $W\prec \mathfrak {A}$ with $\gamma =W \cap \kappa ^+$, which is possible because *T* is stationary. Since $\gamma \in T \cap D$, there is an increasing sequence $\vec {\beta } = \langle {\beta _\alpha }~\vert ~{\alpha < \kappa }\rangle$ that is cofinal in $\gamma$ and has the property that every proper initial segment of $\vec {\beta }$ is equal to $z_\alpha$ for some $\alpha < \gamma$. Since $\vec {z} \in W$ and $W \cap \kappa ^+ =\gamma$, it follows that every proper initial segment of $\vec {\beta }$ is an element of *W*. Recursively define a sequence $\vec {N} = \langle {N_\alpha }~\vert ~{\alpha < \kappa }\rangle$ as follows:

- Given $\alpha <\kappa$, let $N_{\alpha +1}$ be the $\lhd$-least element of $[\mathrm{{H}}(\theta )]^{{<}\kappa }$ such that $N_{\alpha +1}$ is closed under *F*, $\langle {N_\ell }~\vert ~{\ell \le \alpha }\rangle \in N_{\alpha +1}$, $\alpha \subseteq N_{\alpha +1}$, and $\sup (N_{\alpha +1} \cap \kappa ^+) \ge \beta _\alpha$.
- If $\alpha <\kappa$ is a limit ordinal, then $N_\alpha = \bigcup \{{N_\ell }~\vert ~{\ell <\alpha }\}$.

Set $N= \bigcup \{{N_\alpha }~\vert ~{\alpha < \kappa }\}$. Then $N \in \text {IA}_\kappa$, *N* is closed under *F*, and

1 $$\begin{aligned} \sup (N \cap \kappa ^+) ~ \ge ~ \sup _{\alpha < \kappa } \beta _\alpha ~ = ~ \gamma . \end{aligned}$$

On the other hand, for each $\alpha < \kappa$, the sequence $\langle {N_\ell }~\vert ~{\ell \le \alpha }\rangle$ is definable in $\mathfrak {A}$ from the parameter $\langle {\beta _\ell }~\vert ~{\ell \le \alpha }\rangle$, which is an element of *W* by the above remarks. Hence every proper initial segment of $\vec {N}$ is an element of *W* and, in particular, we know that

$$\begin{aligned} \sup (N_\alpha \cap \kappa ^+) ~ < ~ \gamma ~ = ~ W \cap \kappa ^+ \end{aligned}$$

for all $$\alpha < \kappa $$.

It follows that $\sup (N \cap \kappa ^+) \le \gamma$. Combined with (1), this shows that $\sup (N \cap \kappa ^+) = \gamma$. Finally, since $\alpha \subseteq N_{\alpha +1}$ for all $\alpha < \kappa$, it follows that $\kappa \subseteq N$ and hence we know that $N \cap \kappa ^+$ is transitive. This allows us to conclude that

$$\begin{aligned} N \cap \kappa ^+ ~ = ~ \sup (N \cap \kappa ^+) ~ = ~ \gamma , \end{aligned}$$

completing the proof of the lemma. $\square$

The following lemma is one way to salvage stationary set preservation in the non-GCH context.

### Lemma 2.12

Let ${\mathbb {P}}$ be a $\text {IA}_\kappa$-proper poset and let $T \subseteq S^{\kappa ^+}_\kappa$ be stationary with $T \in I[\kappa ^+]$. Then forcing with ${\mathbb {P}}$ preserves the stationarity of *T*.

### Proof

Set $\tau = \kappa ^+$. Let $\dot{C}$ be a ${\mathbb {P}}$-name for a club in $\tau$, let $p \in \mathbb {P}$ and let $\theta$ be a sufficiently large regular cardinal. Using Lemma 2.11, we find $\gamma \in T$ and $W \prec (\mathrm{{H}}(\theta ),\in ,p,\dot{C},{\mathbb {P}})$ with $W \in \text {IA}_\kappa$ and $W \cap \tau =\gamma$. By our assumptions, there is a $(W,{\mathbb {P}})$-master condition *q* below *p* in ${\mathbb {P}}$. Let *G* be ${\mathbb {P}}$-generic over $\mathrm{{V}}$ with $q\in G$. Then $W[G]\cap \tau =W\cap \tau =\gamma$. Moreover, since $\dot{C} \in W$, we now know that $\dot{C}^G\cap W[G]$ is unbounded in $\gamma$ and hence $\gamma \in \dot{C}^G\cap T$.

These computations show that, in the ground model $\mathrm{{V}}$, we have

$$\begin{aligned} q\Vdash _{\mathbb {P}}{\text {``}{\dot{C}\cap \check{T}\ne \emptyset }\text {''}} \end{aligned}$$

for densely-many conditions *q* in ${\mathbb {P}}$. $\square$

## Generalizing a lemma of Jensen

The notion of $\text {IA}_\kappa$-properness, defined in Sect. 2, is a non-$\mathrm{{GCH}}$ analogue of the notion of $\kappa$-*properness* introduced in [13,  Definition 3.4] . This notion will be important to proving that tails of the iteration described in Sect. 4 do not add cofinal branches to a certain tree, and that argument will closely follow the corresponding arguments of [13].

However, $\text {IA}_\kappa$-properness (in the case $\kappa = \omega _1$) is **not** sufficient for ensuring the preservation of forcing axioms that we need for the proofs of our main results. There are examples of $\text {IA}_{\omega _1}$-proper forcings that destroy, for example, the Proper Forcing Axiom.^6^ On the other hand, ${<}\omega _2$-directed closed posets preserve all standard forcing axioms (see [17] and [18]). In this section, we generalize a result of Jensen, yielding a property that is forcing equivalent to ${<}\omega _2$-directed closure, but often easier to verify than ${<}\omega _2$-directed closure.

In [16], Jensen defines a poset ${\mathbb {P}}$ to be **complete** if for every sufficiently large $\theta$, there are club-many $W \in {\wp }_{\omega _1}(\mathrm{{H}}(\theta ))$ such that every $(W,{\mathbb {P}})$-generic filter has a lower bound in ${\mathbb {P}}$.^7^ He then proves:

### Lemma 3.1

(Jensen) The following statements are equivalent for every poset ${\mathbb {P}}$:

1. The poset ${\mathbb {P}}$ is complete.
2. The poset ${\mathbb {P}}$ is forcing equivalent to a $\sigma$-closed poset.

We will generalize a version of this lemma to larger cardinals, and, in fact, characterize directed closure (see Lemma 3.6 below).^8^ However there are a few technicalities to address. Note that for any $W \in {\wp }_{\omega _1}(\mathrm{{H}}(\theta ))$, the fact that *W* is countable ensures that there always exist $(W,{\mathbb {P}})$-generic filters, regardless of what ${\mathbb {P}}$ is. In particular, the phrase “$\dots$ *every* $(W,{\mathbb {P}})$*-generic filter * ...” is never vacuous, if *W* is countable. Of course, for uncountable *W*, it may happen that (depending on the poset ${\mathbb {P}}$) there do not exist any $(W,{\mathbb {P}})$-generic filters at all; e.g. if $W \prec \mathrm{{H}}(\theta )$ and $\omega _1 \subseteq W$, then there does not exist a $(W,\mathrm{{Col}}({\omega },{\omega _1}))$-generic filter.

### Definition 3.2

Given a regular uncountable cardinal $\tau$, a poset ${\mathbb {P}}$ is $\varvec{\tau }$**-Jensen-complete** if the following statements hold for all sufficiently large regular cardinals $\theta$:

1. For every $p \in {\mathbb {P}}$, there are stationarily-many $W \in {\wp }^*_{\tau }(\mathrm{{H}}(\theta ))$ with the property that there exists a $(W,{\mathbb {P}})$-generic filter including *p*.
2. For all but non-stationarily many $W \in {\wp }^*_{\tau }(\mathrm{{H}}(\theta ))$, every $(W,{\mathbb {P}})$-generic filter has a lower bound in ${\mathbb {P}}$.^9^

### Remark 3.3

Note that clause (1) of Definition 3.2 always holds true for $\tau = \omega _1$, and is hence redundant in that case. In particular, for $\tau = \omega _1$, Definition 3.2 is equivalent to Jensen’s definition of completeness.

### Remark 3.4

In combination, the clauses (1) and (2) of Definition 3.2 imply that the poset ${\mathbb {P}}$ is *totally proper* on a stationary subset of ${\wp }^*_{\tau }(\mathrm{{H}}(\theta ))$; i.e. that there are stationarily-many $W \in {\wp }^*_{\tau }(\mathrm{{H}}(\theta ))$ such that every condition in ${\mathbb {P}}\cap W$ can be extended to a $(W,{\mathbb {P}})$-total master condition in the sense of Definition 2.1. This conclusion, however, is strictly weaker than $\tau$-Jensen-completeness, since (for example with $\tau = \omega _1$) shooting a club through a bistationary subset of $\omega _1$ has the latter property but is not ${<}\omega _1$-closed. In the case $\tau = \omega _2$, if $2^{\omega _1} = \omega _2$, then shooting an $\omega _1$-club through the set *M* described in Lemma 2.10 is $\text {IA}_{\omega _1}$-totally proper, but forces the approachability property to hold at $\omega _2$. In particular, this forcing destroys the Proper Forcing Axiom, and $\mathrm{{PFA}}$ is preserved by $\omega _2$-Jensen-complete forcings (by [17] and Lemma 3.6 below).

### Lemma 3.5

If $\kappa$ is an infinite cardinal and ${\mathbb {P}}$ is a ${<}\kappa$-closed poset, then clause (1) of Definition 3.2 holds for $\tau = \kappa ^+$ and ${\mathbb {P}}$.

### Proof

This follows immediately from Lemmas 2.6 and 2.7. $\square$

Next, we state our generalization of Jensen’s lemma. Its Corollary 2 will be used in the proof of Theorem 4.2 below.

### Lemma 3.6

Given a poset $\mathbb {P}$ and a successor cardinal $\tau$, the following statements are equivalent:

1. The poset ${\mathbb {P}}$ is forcing equivalent to a ${<}\tau$-directed closed poset.
2. The poset ${\mathbb {P}}$ is forcing equivalent to a $\tau$-Jensen-complete poset.

### Proof

First, assume that ${\mathbb {P}}$ is ${<}\tau$-directed closed. Then, in particular, ${\mathbb {P}}$ is ${<}\tau$-closed, and hence Lemma 3.5 ensures that clause (1) of Definition 3.2 holds for ${\mathbb {P}}$. But then the directed closure of ${\mathbb {P}}$ ensures that *any* $(W,{\mathbb {P}})$-generic filter for *any* $W \in {\wp }^*_{\tau }(\mathrm{{H}}(\theta ))$ has a lower bound in ${\mathbb {P}}$, and hence clause (2) of Definition 3.2 holds for ${\mathbb {P}}$ as well. This shows that ${\mathbb {P}}$ is $\tau$-Jensen-complete.

Now, suppose that ${\mathbb {P}}$ is $\tau$-Jensen-complete. Let ${F}:{[\mathrm{{H}}(\theta )]^{{<}\omega }}\longrightarrow {\mathrm{{H}}(\theta )}$ generate a club witnessing clause (2) of Definition 3.2, i.e. whenever $W \in {\wp }^*_{\tau }(\mathrm{{H}}(\theta ))$ and *W* is closed under *F*, then any $(W,{\mathbb {P}})$-generic filter has a lower bound. We may assume that *F* also codes a well-ordering $\lhd$ of $\mathrm{{H}}(\theta )$, i.e. if *W* is closed under *F*, then $W \prec (\mathrm{{H}}(\theta ),\in ,\lhd )$) holds.

Define a poset ${\mathbb {Q}}$, whose conditions are pairs (*M*, *g*) satisfying the following statements:

- $$M \in {\wp }^*_{\tau }(\mathrm{{H}}(\theta ))$$.
- *M* is closed under *F*.
- $g \subseteq M \cap {\mathbb {P}}$ is an $(M,{\mathbb {P}})$-generic filter.

and whose ordering is given by:Note that $\le _{\mathbb {Q}}$ is transitive and clause (1) of Definition 3.2 ensures that ${\mathbb {Q}}$ is nonempty.

### Claim 3.7

$\mathbb {Q}$ is ${<}\tau$-directed closed.

### Proof of Claim 3.7

Let $\{{(M_i,g_i)}~\vert ~{i \in I}\}$ be a directed set of conditions in ${\mathbb {Q}}$ with $\vert {I}\vert <\tau$. Set $M= \bigcup _{i \in I} M_i$ and $g=\bigcup _{i \in I} g_i$. We will show that (*M*, *g*) is a condition in ${\mathbb {Q}}$ below all $(M_i,g_i)$.

The regularity of $\tau$ ensures that $M \in {\wp }^*_{\tau }(\mathrm{{H}}(\theta ))$, and *M* is closed under *F*, because each $M_i$ is closed under *F* and the collection $\langle {M_i}~\vert ~{i \in I}\rangle$ is $\subseteq$-directed. In addition, we have $g\subseteq M\cap {\mathbb {P}}$ and *g* clearly has the property that $D \cap g \ne \emptyset$ for every dense $D \in M$, because each such *D* lies in some $M_i$ and $g_i\subseteq g$ is an $(M_i,{\mathbb {P}})$-generic filter. Finally, the fact that *g* is a filter follows easily from the fact that the given collection is directed and each $g_i$ is a filter. This shows that (*M*, *g*) is a condition in ${\mathbb {Q}}$.

Now, fix $i \in I$. Then $M \supseteq M_i$, $g \cap M_i$ is a filter on $M_i \cap {\mathbb {P}}$, and $g \cap M_i\supseteq g_i$. But since $g_i$ is $(M_i,{\mathbb {P}})$-generic, we know that $g_i$ is a $\subseteq$-maximal filter on $M_i \cap {\mathbb {P}}$. In particular, we can conclude that $g \cap M_i = g_i$. This computation shows that $(M,g)\le _{\mathbb {Q}}(M_i,g_i)$. $\square$

### Claim 3.8

The poset ${\mathbb {Q}}$ is forcing equivalent to ${\mathbb {P}}$.

### Proof of Claim 3.8

It is easy to see that the boolean completions of $\tau$-Jensen-complete posets are themselves $\tau$-Jensen-complete. Therefore, we may assume that ${\mathbb {P}}$ is a complete boolean algebra. For each condition (*M*, *g*) in ${\mathbb {Q}}$, let $p_{M,g}$ be the ${\mathbb {P}}$-greatest lower bound of *g*. This conditions exists and is non-zero, because *M* is closed under *F*, *g* is $(M,{\mathbb {P}})$-generic, ${\mathbb {P}}$ is a complete boolean algebra, and because of clause (2) of Definition 3.2. In the following, we will show that the map

$$\begin{aligned} {e}:{{\mathbb {Q}}}\longrightarrow {{\mathbb {P}}};~{(M,g)}\longmapsto {p_{M,g}} \end{aligned}$$

is a dense embedding, which will finish the proof of the claim.

First, we show that *e* is order-preserving. Suppose that $(N,h) \le _{\mathbb {Q}}(M,g)$. Then $N \supseteq M$ and $g = h \cap M$. Since $g \subseteq h$ and $p_{N,h}$ is a lower bound of *h*, it follows that $p_{N,h}$ is also a lower bound of *g*. But $p_{M,g}$ is the greatest lower bound of *g*, and hence

$$\begin{aligned} e(N,h) ~ = ~ p_{N,h} ~ \le _{\mathbb {P}}~ p_{M,g} ~ = ~ e(M,g). \end{aligned}$$

Next, we show that *e* preserves incompatibility. Suppose $(M_0,g_0)$ and $(M_1,g_1)$ are conditions in ${\mathbb {Q}}$ with the property that there is a condition *p* in ${\mathbb {P}}$ that extends both $e(M_0,g_0)$ and $e(M_1,g_1)$. By clause (1) of Definition 3.2, there we can find $W \in {\wp }^*_{\tau }(\mathrm{{H}}(\theta ))$ such that $p,g_0, g_1,M_0,M_1\in W$, *W* is closed under *F*, and there exists a $(W,{\mathbb {P}})$-generic filter *G* with $p \in G$. Since $W \cap \tau$ is transitive and $\vert {M_i}\vert < \tau$ for all $i<2$, it follows that $M_0\cup M_1 \subseteq W$. Furthermore, since *p* is below both $p_{M_0,g_0}$ and $p_{M_1,g_1}$ and $M_i \cap {\mathbb {P}}\subseteq W \cap {\mathbb {P}}$ for all $i<2$, the fact that $g_0$ and $g_1$ are maximal filters in $M_0\cap {\mathbb {P}}$ and $M_1\cap {\mathbb {P}}$, respectively, implies that $G \cap M_0 = g_0$ and $G \cap M_1 = g_1$. Hence (*W*, *G*) is a condition in ${\mathbb {Q}}$ that lies below both $(M_0,g_0)$ and $(M_1,g_1)$.

Finally, we show that the range of *e* is dense in ${\mathbb {P}}$. Fix a condition *p* in ${\mathbb {P}}$. By clause (1) of Definition 3.2, there is a $W \in {\wp }^*_{\tau }(\mathrm{{H}}(\theta ))$ such that *W* is closed under *F*, and there exists a $(W,{\mathbb {P}})$-generic filter *G* with $p \in G$. Then (*W*, *G*) is a condition in ${\mathbb {Q}}$, and $e(W,g)=p_{W,G}$ is stronger than *p*. $\square$

This completes the proof of the lemma. $\square$

### Corollary 1

Given a successor cardinal $\tau$, all $\tau$-Jensen-complete posets are ${<}\tau$-distributive. $\square$

### Remark 3.9

Another common way to verify the ${<}\tau$-distributivity of a given poset ${\mathbb {P}}$ is the following weaker version of $\tau$-Jensen completeness: if for every $p \in {\mathbb {P}}$, there are stationarily-many $W \in {\wp }^*_{\tau }(\mathrm{{H}}(\theta ))$ such that there is a $(W,{\mathbb {P}})$-*total* master condition below *p* (see Definition 2.1), then ${\mathbb {P}}$ is ${<}\tau$-distributive. Note that this weaker version would not suffice for our purposes, however, because we seem to need ${<}\tau$-directed closure (or a close approximation of it) to prove Theorem 4.2.

### Corollary 2

Let $\kappa$ be an infinite regular cardinal and set $\tau = \kappa ^+$. If ${\mathbb {P}}$ is ${<}\kappa$-closed poset with the property that for all but non-stationarily many $W \in {\wp }^*_{\tau }(\mathrm{{H}}(\theta ))$, every $(W,{\mathbb {P}})$-generic filter has a lower bound in ${\mathbb {P}}$, then the poset ${\mathbb {P}}$ is forcing equivalent to a ${<}\tau$-directed closed poset.

### Proof

By Lemma 3.5, the ${<}\kappa$-closure of ${\mathbb {P}}$ ensures that clause (1) of Definition 3.2 holds. Since clause (2) of Definition 3.2 holds by assumption, this implies $\mathbb {P}$ is $\tau$-Jensen-complete and Lemma 3.6 yields the desired conclusion. $\square$

In particular, if a poset ${\mathbb {P}}$ satisfies the assumptions of the above corollary for $\kappa =\omega _1$, then forcing with ${\mathbb {P}}$ preserves all standard forcing axioms.

## The main technical result

In this section, we will prove the main technical result of our paper. It directly extends the main results of [13] and [23]. In the next section, we will use it to prove the two theorems stated in the introduction.

### Definition 4.1

If $\kappa$ be an infinite regular cardinal and let $S\subseteq S^{\kappa ^+}_\kappa$. Then we let *T*(*S*) denote the tree that consists of all $t\in {}^{{<}\kappa ^+}\kappa ^+$ such that ${\mathrm{{dom}}(t)}$ is a successor ordinal, ${\mathrm{{ran}}(t)}\subseteq S$, *t* is strictly increasing, and *t* is continuous at all points of cofinality $\kappa$ in its domain and is ordered by end-extension.

Note that, in the situation of the above definition, the tree *T*(*S*) has height $\kappa ^+$ and contains a cofinal branch if and only if the set *S* contains a $\kappa$-club.

We are now ready to state the aspired result.

### Theorem 4.2

Given an infinite regular cardinal $\kappa$, there is a partial order ${\mathbb {P}}$ with the following properties:

1. ${\mathbb {P}}$ is $\kappa ^+$-Jensen complete.^10^
2. ${\mathbb {P}}$ satisfies the $(2^\kappa )^+$-chain condition.
3. If *G* is ${\mathbb {P}}$-generic over $\mathrm{{V}}$, then, in *V*[*G*], there is a subtree *T* of ${}^{{<}\kappa ^+}\kappa ^+$ of height $\kappa ^+$ without cofinal branches such that the following statements hold:
  - If *S* is bistationary in $S^{\kappa ^+}_\kappa$ and $S^{\kappa ^+}_\kappa \setminus S$ contains a stationary set in $I[\kappa ^+]$, then there is an order-preserving function from *T*(*S*) to *T*.
  - Assume that $\kappa ^{{<}\kappa } \le \kappa ^+$ holds in $\mathrm{{V}}$. If $M\in \mathrm{{V}}$ is a maximum element of $I[\kappa ^+]\cap {\wp }({S^{\kappa ^+}_\kappa })$ mod ${{NS}}_{}$ in $\mathrm{{V}}$,^11^ then the following statements hold in $\mathrm{{V}}[G]$:
    (i) *M* is a maximum element of $I[\kappa ^+] \cap {\wp }({S^{\kappa ^+}_\kappa })$ mod ${{NS}}_{}$.
    (ii) If *S* is a bistationary in $S^{\kappa ^+}_\kappa$ and $M \setminus S$ is stationary, then there is an order-preserving function from *T*(*S*) to *T*.

Note that the above theorem directly generalizes the main result of [13]: if $\kappa ^{{<}\kappa }=\kappa$ holds, then part (4) of Lemma 2.10 shows that $S^{\kappa ^+}_\kappa$ is an element of $I[\kappa ^+]$, and hence $S^{\kappa ^+}_\kappa$ is a maximum element of $I[\kappa ^+]\cap {\wp }({S^{\kappa ^+}_\kappa })$ mod ${{NS}}_{}$.

Now, if *G* is ${\mathbb {P}}$-generic over $\mathrm{{V}}$ and $T\in \mathrm{{V}}[G]$ is the tree given by the theorem, then there is an order-preserving function from the tree *T*(*S*) to *T* in $\mathrm{{V}}[G]$ for every bistationary subset *S* of $S^{\kappa ^+}_\kappa$ in $\mathrm{{V}}[G]$.

In particular, this shows that *T* is a $\kappa$-*canary tree* (see [13,  Definition 3.1]) in $\mathrm{{V}}[G]$, i.e. if *S* is a stationary subset of $S^{\kappa ^+}_\kappa$ and ${\mathbb {P}}$ is a ${<}\kappa ^+$-distributive poset that forces $\kappa ^+\setminus S$ to contain a club subset, then forcing with ${\mathbb {P}}$ adds a cofinal branch to *T*.

For the remainder of this section, fix an infinite regular cardinal $\kappa$. Until further notice, we do **not** make any cardinal arithmetic assumptions.

In the following, we closely follow the arguments on pages 1684–1692 of Hyttinen-Rautila in [13], which assumed $\mathrm{{GCH}}$ (in particular, their arguments heavily rely on the assumption $\kappa ^{{<}\kappa }=\kappa$). We also follow their notation as closely as possible.

### Definition 4.3

( [13]) We let ${\mathbb {Q}}_0$ denote the poset that consists of functions *f* such that ${\mathrm{{dom}}(f)}\subseteq S^{\kappa ^+}_\kappa$, $\vert {{\mathrm{{dom}}(f)}}\vert \le \kappa$, $f(\delta )$ is a function from $\delta$ to $\delta$ for all $\delta \in {\mathrm{{dom}}(f)}$, and whenever $\delta < \eta$ are both in the domain of *f*, then $f(\delta ) \nsubseteq f(\eta )$,^12^ and whose ordering is given by reversed inclusion.

### Proposition 4.4

The poset ${\mathbb {Q}}_0$ is ${<}\kappa ^+$-directed closed.

### Proof

This statement follows directly from the fact that the union *f* of a coherent collection of conditions in ${\mathbb {Q}}_0$ still has the required property that $f(\eta ) \nsubseteq f(\beta )$ for all $\eta < \beta$ in the domain of *f*, and, if the union is of size less than $\kappa ^+$, then the domain of *f* has size less than $\kappa ^+$ too. $\square$

### Definition 4.5

( [13]) If $G_0$ is ${\mathbb {Q}}_0$-generic over $\mathrm{{V}}$, then, in $\mathrm{{V}}[G_0]$, we define the following subtree of ${}^{{<}\kappa ^+} \kappa ^+$:

$$\begin{aligned} \mathcal {T}(G_0) ~ = ~ \{{h \in {}^{{<}\kappa ^+} \kappa ^+}~\vert ~{\forall \delta \in S^{\kappa ^+}_\kappa ~ h\restriction \delta \ne ({\textstyle \bigcup G_0})(\delta )}\}. \end{aligned}$$

In the following, we let $\dot{\mathcal {T}}(\dot{G}_0)$ denote the canonical ${\mathbb {Q}}_0$-name for $\mathcal {T}(G_0)$.

### Remark 4.6

In the situation of the above definition, the tree $\mathcal {T}(G_0)$ has height $\kappa ^+$, since for any $\beta \in S^{\kappa ^+}_\kappa$, the function *f* with domain $\beta$ and constant value $\beta + 1$ has the property that for all $\delta \in S^{\kappa ^+}_\kappa$ with $\delta \le {\mathrm{{dom}}(f)}$, the restriction $f\restriction \delta$ is not a function from $\delta$ to $\delta$ and hence cannot be the same as the function $(\bigcup G_0)(\delta )$.

### Lemma 4.7

If $G_0$ is ${\mathbb {Q}}_0$-generic over $\mathrm{{V}}$, then the tree $\mathcal {T}(G_0)$ has no cofinal branches in $\mathrm{{V}}[G_0]$.

### Proof

Work in $\mathrm{{V}}$ and assume, towards a contradiction, that a condition *f* in ${\mathbb {Q}}_0$ forces a ${\mathbb {Q}}_0$-name $\dot{b}$ to be a cofinal branch through $\dot{\mathcal {T}}(\dot{G}_0)$. Using Proposition 4.4, easy closure arguments allow us to find $\lambda \in S^{\kappa ^+}_\kappa$, a function ${h}:{\lambda }\longrightarrow {\lambda }$ and a condition *g* below *f* in ${\mathbb {Q}}_0$ such that $\lambda =\sup ({\mathrm{{dom}}(g)})$ and *g* forces *h* to be the restriction of $\dot{b}$ to $\lambda$. By the definition of $\mathcal {T}(G_0)$, this implies that $h\restriction \delta \ne g(\delta )$ holds for all $\delta \in {\mathrm{{dom}}(g)}$ and we can conclude that $g\cup \{(\lambda ,h)\}$ is a condition in ${\mathbb {Q}}_0$ below *g*. But this condition forces that *h* is not contained in $\dot{\mathcal {T}}(\dot{G}_0)$, a contradiction. $\square$

The following poset, again taken from [13], adds an order preserving function from *T*(*S*) to $\mathcal {T}(G_0)$. The role of clause 3(a)i is to add such a function with initial segments. However, the role of clauses 3(a)ii through 3(a)vi is not obvious; roughly, with the exception of clause 3(a)iv, these properties allow us to verify $\kappa ^+$-Jensen completeness by ensuring the existence of lower bounds for any generic filter over any $\kappa$-sized elementary submodel. The role of clause 3(a)iv is to ensure that no cofinal branch is added to $\mathcal {T}(G_0)$.

### Definition 4.8

([13]) Let $G_0$ be ${\mathbb {Q}}_0$-generic over $\mathrm{{V}}$ and work in an outer model of $\mathrm{{V}}[G_0]$^13^ with the same bounded subsets of $\kappa ^+$ as $\mathrm{{V}}$. Let *S* be a subset of $S^{\kappa ^+}_\kappa$.

1. An element *t* of $\mathcal {T}(G_0)$ is an $\varvec{S}$**-node** if $t[\delta ]\nsubseteq \delta$ holds for all $\delta \in S^{\kappa ^+}_\kappa \setminus S$.
2. Given a partial function ${h}:{T(S)}\xrightarrow {part}{ \mathcal {T}(G_0)}$, we define
  $$\begin{aligned} o(h) ~ = ~ \sup \{{{\mathrm{{dom}}(t)}}~\vert ~{t\in {\mathrm{{ran}}(h)}}\}. \end{aligned}$$
3. We let ${\mathbb {P}}(S,G_0)$ denote the unique poset defined by the following clauses:
  - A condition in ${\mathbb {P}}(S,G_0)$ is a pair (*h*, *X*) satisfying the following statements:
    (i) *h* is an order-preserving partial function of cardinality at most $\kappa$ from the tree *T*(*S*) to the tree $\mathcal {T}(G_0)$ with the property that ${\mathrm{{dom}}(h)}$ is closed under initial segments.
    (ii) *X* is a partial function from $\kappa ^+$ to $\bigcup \{{{}^{\beta +1}\kappa ^+}~\vert ~{\beta < \kappa ^+}\}$ of cardinality at most $\kappa$ such that
      $$\begin{aligned} o(h) \cap S^{\kappa ^+}_\kappa \subseteq {\mathrm{{dom}}(X)} \end{aligned}$$
      and
      $$\begin{aligned} X(\alpha ) \subseteq ( {\textstyle \bigcup G_0})(\alpha ) \end{aligned}$$
      for all $$\alpha \in {\mathrm{{dom}}(X)}\cap S^{\kappa ^+}_\kappa $$.
    (iii) ${\mathrm{{dom}}(h(t))}= \sup ({\mathrm{{ran}}(t)})$ for all $t \in {\mathrm{{dom}}(h)}$.
    (iv) *h*(*t*) is an *S*-node for all $t \in {\mathrm{{dom}}(h)}$.
    (v) $X(\alpha ) \nsubseteq h(t)$ for all $t\in {\mathrm{{dom}}(h)}$ and $\alpha \in {\mathrm{{dom}}(X)}$.
    (vi) If $\langle {t_\zeta }~\vert ~{\zeta < \kappa }\rangle$ is a strictly increasing sequence of elements of ${\mathrm{{dom}}(h)}$, then $\bigcup _{\zeta < \kappa } h(t_\zeta ) \in \mathcal {T}(G_0)$.
  - A condition (*h*, *X*) is stronger than a condition (*k*, *Y*) if and only if $k \subseteq h$ and $Y \subseteq X$ hold.
4. The **order** of a condition $p=(h,X)$ in ${\mathbb {P}}(S,G_0)$, denoted by *o*(*p*), is defined to be the ordinal
  $$\begin{aligned} \max \{ \textstyle {\bigcup {\mathrm{{dom}}(X)}}, ~ \textstyle {\bigcup } \{{{\mathrm{{dom}}(h(t))}}~\vert ~{t\in {\mathrm{{dom}}(h)}}\}\}. \end{aligned}$$

### Remark 4.9

In the above definition, the requirements on $X(\alpha )$ differ depending on whether or not ${\mathrm{{cof}}(\alpha )}=\kappa$. If $\alpha \in S^{\kappa ^+}_\kappa \cap {\mathrm{{dom}}(X)}$, then $X(\alpha )$ is a proper initial segment of the function $(\bigcup G_0)(\alpha )$.^14^ In combination with requirement (3(a)v) in the above definition, this shows that for all $\alpha \in {\mathrm{{dom}}(X)}\cap S^{\kappa ^+}_\kappa$, there is an ordinal $\eta _\alpha <\alpha$ such that no node in the range of *h* can extend $(\bigcup G_0)(\alpha )\restriction \eta _\alpha$. On the other hand, if $\alpha \in {\mathrm{{dom}}(X)}$ with ${\mathrm{{cof}}(\alpha )}<\kappa$, then the only requirement on $X(\alpha )$ is that nothing in the range of *h* is allowed to extend $X(\alpha )$.

As pointed out near the bottom of page 1684 of [13], the poset ${\mathbb {P}}(S,G_0)$ is ${<}\kappa$-closed. Requirements (3(a)iii) and (3(a)vi) of Definition 4.8 are mainly needed for the proof of Lemma 4.10 below.

### Lemma 4.10

Let $G_0$ be ${\mathbb {Q}}_0$-generic over $\mathrm{{V}}$, let $\mathrm{{V}}_1$ be an outer model of $\mathrm{{V}}[G_0]$ with the same bounded subsets of $\kappa ^+$ as $\mathrm{{V}}$, and let *K* be ${\mathbb {P}}(S,G_0)^{\mathrm{{V}}_1}$-generic over $\mathrm{{V}}_1$ for some set *S* that is bistationary in $S^{\kappa ^+}_\kappa$ in $\mathrm{{V}}_1$. In $\mathrm{{V}}_1[K]$, define

$$\begin{aligned} h_K ~ = ~ \bigcup \{{h}~\vert ~{(h,X) \in K}\} \end{aligned}$$

and

$$\begin{aligned} X_K ~ = ~ \bigcup \{{X}~\vert ~{(g,X)\in K}\}. \end{aligned}$$

Let $\delta =(\kappa ^+)^\mathrm{{V}}$. Then the following statements hold:

1. $h_K$ is a total, order-preserving function from $(T(S))^{\mathrm{{V}}_1}$ to $(\mathcal {T}(G_0))^{\mathrm{{V}}[G_0]}$ whose range consists entirely of *S*-nodes.
2. $X_K$ is a total function from $\delta$ to $({}^{{<}\delta }\delta )^\mathrm{{V}}$.
3. No element of ${\mathrm{{ran}}(h_K)}$ extends an element of ${\mathrm{{ran}}(X_K)}$.
4. Suppose *M* is an outer model of $\mathrm{{V}}_1[K]$ and $\vec {y}=\langle {y_\zeta }~\vert ~{\zeta <\lambda }\rangle$ is an increasing sequence of nodes in ${\mathrm{{ran}}(h_K)}$ in *M*. If we set $f_{\vec {y}}=\bigcup _{\zeta < \lambda } y_\zeta$, then
  $$\begin{aligned} f_{\vec {y}} \restriction \gamma ~ \ne ~ (\textstyle {\bigcup G_0})(\gamma ) \end{aligned}$$
  for all $$\gamma \in (S^\delta _\kappa )^\mathrm{{V}}$$.

### Proof

Statement (1) is [13,  Claim 3.11].^15^

Statement (2) is an easy density argument, and statement (3) follows directly from requirement 3(a)v of Definition 4.8.

For Statement (4), let *M* and $\vec {y} \in M$ be as stated, and suppose for a contradiction there exists $\gamma <\delta$ with ${\mathrm{{cof}}(\gamma )}^\mathrm{{V}}=\kappa$ and $f_{\vec {y}} \restriction \gamma = (\textstyle {\bigcup G_0})(\gamma )$.

Work in *M*. The statements (1), (2), and (3) are obviously upward absolute from $\mathrm{{V}}_1[K]$ to *M*. By Statement (2), we know that $\gamma$ is in the domain of $X_K$, and, by applying Remark 4.9 to some condition in *K* whose second coordinate has $\gamma$ in its domain, we can find $\rho _\gamma < \gamma$ with $X_K(\gamma ) = (\textstyle {\bigcup G_0})(\gamma ) \restriction \rho _\gamma$. Note that, by the definition of ${\mathbb {Q}}_0$, we know that $(\bigcup G_0)(\gamma )$ is a total function on $\gamma$, and therefore our assumption implies that $\gamma \le {\mathrm{{dom}}(f_{\vec {y}})}$. Then $\rho _\gamma \in {\mathrm{{dom}}(f_{\vec {y}})}$, and there is some $\zeta _*<\lambda$ with $\rho _\gamma \in {\mathrm{{dom}}(y_{\zeta _*})}$. In particular, we know that

$$\begin{aligned} y_{\zeta _*} \restriction \rho _\gamma ~ = ~ f_{\vec {y}} \restriction \rho _\gamma ~ = ~ (\textstyle {\bigcup G_0})(\gamma )\restriction \rho _\gamma ~ = ~ X_K(\gamma ). \end{aligned}$$

But this implies that $y_{\zeta _*}\in {\mathrm{{ran}}(h_K)}$ extends $X_K(\gamma )\in {\mathrm{{ran}}(X_K)}$, contradicting Statement (3). $\square$

We now describe the iteration that will witness the poset from Theorem 4.2. This is a slight variant of the iteration described at the bottom of page 1684 of [13].

The main differences are:

- The length of our iteration is at least $2^{2^\kappa }$. This is to allow for the case when, in the ground model, the cardinal $2^{\kappa ^+}$ is very large.
- More significantly, at a given stage $\alpha$ of our iteration, when considering the set $\dot{S}_\alpha$ given to us by the bookkeeping device, we only force with the poset ${\mathbb {P}}(\dot{S}_\alpha ,G_0)$ if the statement
  2 $$\begin{aligned} {\text {``}{{S^{\kappa ^+}_\kappa \setminus \dot{S}_\alpha \, contains\, a\, stationary\, set\, in\, I[\kappa ^+]}}\text {''}} \end{aligned}$$
  holds in the corresponding generic extension of the ground model. This will ensure (via an application of Lemma 2.12) that the complements of the $\dot{S}_\alpha$’s remain stationary throughout the iteration, which in turn will be the key to showing that the tree $\mathcal {T}(G_0)$ has no cofinal branch in the final model.

### Remark 4.11

In the $\mathrm{{GCH}}$ setting of [13], requiring (2) to hold is no restriction at all, since in that scenario, this statement holds for **every** set bistationary in $S^{\kappa ^+}_\kappa$.

But, if $\kappa ^{{<}\kappa } > \kappa$ holds, then the requirement (2) seems to be needed in order to prove that the iteration adds no cofinal branch to the tree $\mathcal {T}(G_0)$.

In the following, we fix a cardinal $\varepsilon$ satisfying $\varepsilon ^{2^\kappa }=\varepsilon$.

Let $\mathcal {C}$ denote the set of all partial functions from $\varepsilon$ to $\mathrm{{H}}(\kappa ^+)$ of cardinality at most $\kappa$.

Then our assumptions on $\varepsilon$ imply that $\vert {\mathcal {C}}\vert =\varepsilon$.

Next, let $\mathcal {N}$ denote the set of all partial functions from ${S^{\kappa ^+}_\kappa }\times 2^\kappa$ to $\mathcal {C}$. Again, our assumptions imply that $\vert {\mathcal {N}}\vert =\varepsilon$ and we can pick an $\varepsilon$-to-one surjection ${b}:{\varepsilon }\longrightarrow {\mathcal {N}}$.

### Definition 4.12

We define

$$\begin{aligned} \langle {{\mathbb {P}}_\alpha , \dot{{\mathbb {Q}}}_\xi }~\vert ~{\alpha \le \varepsilon , ~ \xi < \varepsilon }\rangle \end{aligned}$$

to be a ${<}\kappa ^+$-support iteration satisfying the following clauses:

1. $\dot{{\mathbb {Q}}}_0$ is chosen in a canonical way that ensures that the map
  $$\begin{aligned} {i}:{{\mathbb {Q}}_0}\longrightarrow {{\mathbb {P}}_1};~{q}\longmapsto {\langle \check{q}\rangle } \end{aligned}$$
  is an isomorphism.
2. Assume that $\alpha \in [1, \varepsilon )$ has the property that the poset ${\mathbb {P}}_\alpha$ is ${<}\kappa ^+$-distributive and there exists a sequence $\langle {q_{\gamma ,\xi }}~\vert ~{(\gamma ,\xi )\in {\mathrm{{dom}}(b(\alpha ))}}\rangle$ of conditions in ${\mathbb {P}}_\alpha$ such that the following statements hold:
  If $$(\gamma ,\xi )\in {\mathrm{{dom}}(b(\alpha ))}$$, then $${\mathrm{{sprt}}(q_{\gamma ,\xi })}={\mathrm{{dom}}(b(\alpha )(\gamma ,\xi ))}$$.
  If $(\gamma ,\xi )\in {\mathrm{{dom}}(b(\alpha ))}$, $\ell \in {\mathrm{{sprt}}(q_{\gamma ,\xi })}$ and $b(\alpha )(\gamma ,\xi )(\ell )=x$, then $q_{\gamma ,\xi }(\ell )=\check{x}$.
  If we define
  $$\begin{aligned} \dot{B}_\alpha ~ = ~ \{{(\check{\gamma },q_{\gamma ,\xi })}~\vert ~{(\gamma ,\xi )\in {\mathrm{{dom}}(b(\alpha ))}}\}, \end{aligned}$$
  then there exists a ${\mathbb {P}}_\alpha$-name $\dot{S}_\alpha$ for a subset of $S^{\kappa ^+}_\kappa$ such that the following statements hold in $\mathrm{{V}}[G]$ whenever *G* is ${\mathbb {P}}_\alpha$-generic over $\mathrm{{V}}$ and $G_0$ is the induced ${\mathbb {Q}}_0$-generic filter over $\mathrm{{V}}$:
  - $$\dot{{\mathbb {Q}}}_\alpha ^G={\mathbb {P}}(\dot{S}_\alpha ^G,G_0)^{\mathrm{{V}}[G]}$$.
  - If the set $S^{\kappa ^+}_\kappa \setminus \dot{B}_\alpha ^G$ contains a stationary set in $I[\kappa ^+]$, then $\dot{S}_\alpha ^G=\dot{B}_\alpha ^G$.
  - If the set $S^{\kappa ^+}_\kappa \setminus \dot{B}_\alpha ^G$ does not contain a stationary set in $I[\kappa ^+]$, then $\dot{S}_\alpha ^G=\emptyset$.
3. Assume that $\alpha \in [1, \varepsilon )$ has the property that the poset ${\mathbb {P}}_\alpha$ is ${<}\kappa ^+$-distributive and there exists no sequence of conditions in ${\mathbb {P}}_\alpha$ with the properties listed in (2). Then $\dot{S}_\alpha =\emptyset$ and $\dot{{\mathbb {Q}}}_\alpha ^G={\mathbb {P}}(\dot{S}_\alpha ^G,G_0)^{\mathrm{{V}}[G]}$ whenever *G* is ${\mathbb {P}}_\alpha$-generic over $\mathrm{{V}}$ and $G_0$ is the induced ${\mathbb {Q}}_0$-generic filter over $\mathrm{{V}}$.
4. If $\alpha \in [1, \varepsilon )$ has the property that the poset ${\mathbb {P}}_\alpha$ is not ${<}\kappa ^+$-distributive, then $\dot{{\mathbb {Q}}}_\alpha$ is a ${\mathbb {P}}_\alpha$-name for a trivial poset.

### Remark 4.13

We include the cases (2c) and (3) in the above definition of the name $\dot{S}_\alpha$ to simplify notation later on. Note that, since we have $T(\emptyset ) = \emptyset$, conditions in ${\mathbb {P}}(\emptyset , G_0)$ always have trivial first coordinate, and the poset ${\mathbb {P}}(\emptyset , G_0)$ is forcing equivalent to $\mathrm{{Add}}({\kappa ^+},{1})$.

Throughout the rest of this paper, ${\mathbb {P}}$ refers to the poset ${\mathbb {P}}_\varepsilon$. Moreover, in order to conform to the notation from [13], if *G* is ${\mathbb {P}}_\alpha$-generic over $\mathrm{{V}}$ for some $\alpha \le \varepsilon$, then we let $G_0$ denote the induced ${\mathbb {Q}}_0$-generic filter over $\mathrm{{V}}$.

### Definition 4.14

A condition *p* in ${\mathbb {P}}$ is called **flat** if there exists a sequence $\langle {x_\alpha }~\vert ~{i\in {\mathrm{{sprt}}(p)}}\rangle$ with the property that $x_\alpha \in \mathrm{{H}}(\kappa ^+)$ and

$$\begin{aligned} p\restriction \alpha \Vdash _{{\mathbb {P}}_\alpha } {\text {``}{p(\alpha )=\check{x}_\alpha }\text {''}} \end{aligned}$$

hold for all $\alpha \in {\mathrm{{sprt}}(p)}$ with the property that ${\mathbb {P}}_\alpha$ is ${<}\kappa ^+$-distributive.

Just as in [13], the flat conditions turn out to be dense in ${\mathbb {P}}$, as we will see in Lemma 4.20 below.

Although the density of the flat conditions is not needed to prove the $\kappa ^+$-Jensen-completeness of ${\mathbb {P}}$ in Lemma 4.20, it will be crucial for the proofs of the following statements:

- The “tails” of the above iteration are proper with respect to $\text {IA}_\kappa$ (see Lemma 4.27), which in turn is important for the proof that the tree $\mathcal {T}(G_0)$ has no cofinal branches in ${\mathbb {P}}$-generic extensions of $\mathrm{{V}}$ (see Lemma 4.28).
- If $2^\kappa = \kappa ^+$, then ${\mathbb {P}}$ satisfies the $\kappa ^{++}$-chain condition.

The function $p(f_0,g)$ defined in Definition 4.15 below is a natural attempt to form a flat condition out of a $(W,{\mathbb {P}})$-generic filter for some elementary substructure *W* of size $\kappa$.

### Definition 4.15

Suppose $W \prec (\mathrm{{H}}(\theta ),\in ,{\mathbb {P}})$ with $\vert {W}\vert =\kappa \subseteq W$, and $g \subseteq {\mathbb {P}}\cap W$ is a $(W,{\mathbb {P}})$-generic filter in $\mathrm{{V}}$.

1. Set $$g_0=\{{q\in {\mathbb {Q}}_0}~\vert ~{\exists p\in g ~ p(0)=\check{q}}\}$$.^16^
2. Given $0<\alpha \le \varepsilon$, we define
  $$\begin{aligned} g_\alpha ~ = ~ \{{ p \restriction \alpha }~\vert ~{p \in g}\}. \end{aligned}$$
3. Given $0<\alpha < \varepsilon$ with the property that the poset ${\mathbb {P}}_\alpha$ is ${<}\kappa ^+$-distributive, let $\dot{c}_{g,\alpha }$, $\dot{h}_{g,\alpha }$ and $\dot{X}_{g,\alpha }$ denote the canonical ${\mathbb {P}}_\alpha$-names with the property that,^17^ whenever *G* is ${\mathbb {P}}_\alpha$-generic over $\mathrm{{V}}$, then
  $$\dot{c}_{g,\alpha }^G ~ = ~ \{{p(\alpha )^G}~\vert ~{p \in g}\}$$,
  $$\dot{h}_{g,\alpha }^G ~ = ~ \bigcup \{{h}~\vert ~{\exists X ~ (h,X)\in \dot{c}_{g,\alpha }^G}\}$$, and
  $$\dot{X}_{g,\alpha }^G ~ = ~ \bigcup \{{X}~\vert ~{\exists h ~ (h,X)\in \dot{c}_{g,\alpha }^G}\}$$.
4. If $f_0$ is a condition in ${\mathbb {Q}}_0$ extending $\bigcup g_0$, then we define a function $p(f_0, g)$ with domain $W \cap \varepsilon$ by setting
  $$\begin{aligned} p(f_0, g)= & {} \langle f_0 \rangle ^\frown \langle {\mathsf {pair}_{{\mathbb {P}}_\alpha }(\dot{h}_{g,\alpha },\dot{X}_{g,\alpha })}~\vert ~{\alpha }\rangle \in W\\&\cap&[1,\varepsilon )\, with\, {\mathbb {P}}_\alpha {<}\kappa ^+-distributive, \end{aligned}$$
  where $\mathsf {pair}_{{\mathbb {P}}_\alpha }(\dot{h}_{g,\alpha },\dot{X}_{g,\alpha })$ denotes the canonical ${\mathbb {P}}_\alpha$-name for the ordered pair of $\dot{h}_{g,\alpha }$ and $\dot{X}_{g,\alpha }$.

Note that, in the last part of the above definition, the function $p(f_0, g)$ may or may not be a condition in ${\mathbb {P}}$. The following lemma shows how we can ensure that $p(f_0, g)$ is a flat condition below every condition in *g*.

### Lemma 4.16

Suppose *W*, *g*, and $f_0$ are as in Definition 4.15. Then one of the following statements holds:

1. $p(f_0, g)$ is a flat condition in ${\mathbb {P}}$ that extends every element of *g*.
2. There is an $\alpha \in W \cap [1,\varepsilon )$ such that the following statements hold:
  - $p(f_0, g)\restriction \alpha$ is a condition in ${\mathbb {P}}_\alpha$ that extends every element of $g_\alpha$.
  - If $\tau \in W$ is a ${\mathbb {P}}_\alpha$-name for a function from $\kappa$ to the ordinals, then
    $$\begin{aligned} p(f_0,g)\restriction \alpha \Vdash _{{\mathbb {P}}_\alpha }{\text {``}{\tau \in \check{W}}\text {''}}. \end{aligned}$$
  - There is a condition *q* in ${\mathbb {P}}_\alpha$ below $p(f_0,g)\restriction \alpha$ with the property that the following statements hold true in $\mathrm{{V}}[G]$, whenever *G* is ${\mathbb {P}}_\alpha$-generic over $\mathrm{{V}}$ with $q\in G$:
    (i) ${\mathrm{{cof}}(W\cap \kappa ^+)}^\mathrm{{V}}=\kappa$. In particular, we have $W\cap \kappa ^+\in {\mathrm{{dom}}(\bigcup G_0)}$.
    (ii) Every proper initial segment of $(\bigcup G_0)(W\cap \kappa ^+)$ is an element of *W*, and is an $\dot{S}_\alpha ^G$-node.
    (iii) No proper initial segment of $(\bigcup G_0)(W\cap \kappa ^+)$ is an element of ${\mathrm{{ran}}(\dot{X}_{g,\alpha }^G)}$.

### Proof

Set $g_\varepsilon =g$ and, given $\beta \le \varepsilon$, let $\Phi _\beta$ denote the statement asserting that $p(f_0,g)\restriction \beta$ is a flat condition in ${\mathbb {P}}_\beta$ that lies below every element of $g_\beta$.

Suppose that $\Phi _\varepsilon$ fails, i.e. that part (1) of the disjunctive conclusion of the statement of the lemma fails. Let $\beta \le \varepsilon$ be the least ordinal such that $\Phi _\beta$ fails.

### Claim 4.17

$\beta$ is a successor ordinal and an element of *W*.

### Proof of Claim 4.17

First, we have $\beta >0$, because the 0th component of $p(f_0,g)$ is $f_0$, which is assumed to be a condition stronger than $\bigcup g_0$, and $g_0$ is a $(W,{\mathbb {Q}}_0)$-generic filter.

Now, assume, towards a contradiction, that $\beta$ is a limit ordinal.

Since $\Phi _\alpha$ holds for all $\alpha <\beta$ and since the support of $p(f_0,g)$ is contained in the $\kappa$-sized set $W\cap \varepsilon$, it follows easily that $p(f_0,g)\restriction \beta$ is a condition and is below every element of $g_\beta$.

Furthermore, for each $\alpha \in W \cap \beta$, let

$$\begin{aligned} \vec {x}_\alpha ~ = ~ \langle {x^\alpha _\xi }~\vert ~{\xi \in W \cap \alpha }\rangle \end{aligned}$$

witness flatness of $p(f_0,g) \restriction \alpha$. Then for all $\alpha _0<\alpha _1 <\beta$, it follows easily that $x^{\alpha _0}_\xi = x^{\alpha _1}_\xi$ holds for all $\xi \in W \cap \alpha _0$. So the $\vec {x}_\alpha$’s are coherent, and their union witnesses flatness of $p(f_0,g) \restriction \beta$. This shows that $\Phi _\beta$ holds, a contradiction.

The above computations yield an ordinal $\alpha$ with $\beta =\alpha +1$. Assume, towards a contradiction, that $\alpha \notin W$.

Note that, if $r \in g$, then $r \in W$ and, since ${\mathrm{{sprt}}(r)}$ is a $\kappa$-sized element of *W* and $\kappa \subseteq W$, it follows that ${\mathrm{{sprt}}(r)} \subseteq W$.

Since $\alpha$ is not an element of *W*, this shows that $g_\alpha =g_\beta$ and $p(f_0,g)\restriction \beta =p\big (f_0,g)\restriction \alpha$. But, since $\Phi _\alpha$ holds, this immediately implies that $\Phi _\beta$ holds too, a contradiction. $\square$

The above claim shows that there is an $\alpha \in W \cap [1,\varepsilon )$ with $\beta = \alpha + 1$. We claim that this $\alpha$ witnesses part (2) of the conclusion of the lemma holds true. By the minimality of $\beta$, we know that (2a) holds and ${\mathbb {P}}_\alpha$ is ${<}\kappa ^+$-distributive. Moreover, since $p(f_0,g) \restriction \alpha$ is a condition that extends the $(W,{\mathbb {P}}_\alpha )$-generic filter $g_\alpha$, part (2b) holds by Lemma 2.2.

### Claim 4.18

There is an $x \in \mathrm{{H}}(\kappa ^+)$ with

3 $$\begin{aligned} p(f_0,g) \restriction \alpha \Vdash _{{\mathbb {P}}_\alpha }{\text {``}{p(f_0,g)(\alpha )=\check{x}}\text {''}}. \end{aligned}$$

Furthermore, if *G* is ${\mathbb {P}}_\alpha$-generic over $\mathrm{{V}}$ with $p(f_0,g)\restriction \alpha \in G$, then the following statements hold in $\mathrm{{V}}[G]$:

1. The pair $(\dot{h}_{g,\alpha }^G,\dot{X}_{g,\alpha }^G)$ satisfies all requirements to be a condition in the poset ${\mathbb {P}}(\dot{S}_\alpha ^G,G_0)$, **with the possible exception of** requirement (3(a)vi) of Definition 4.8. In particular, the following statements hold:
  - Every element of ${\mathrm{{ran}}(\dot{h}_{g,\alpha }^G)}$ is an $\dot{S}_\alpha ^G$-node (i.e. requirement (3(a)iv) of Definition 4.8 is satisfied).
  - No element of ${\mathrm{{ran}}(\dot{h}_{g,\alpha }^G)}$ extends an element of ${\mathrm{{ran}}(\dot{X}_{g,\alpha }^G)}$ (i.e. requirement (3(a)v) of Definition 4.8 is satisfied).
2. **If** the pair $(\dot{h}_{g,\alpha }^G,\dot{X}_{g,\alpha }^G)$ is **not** a condition in ${\mathbb {P}}(\dot{S}_\alpha ^G,G_0)$, then the following statements hold:
  - ${\mathrm{{cof}}(W \cap \kappa ^+)}^\mathrm{{V}}= \kappa$. In particular, we have $W \cap \kappa ^+\in {\mathrm{{dom}}(\bigcup G_0)}$).
  - Every proper initial segment of $(\bigcup G_0)(W \cap \kappa ^+)$ is an element of *W*, and is an $\dot{S}_\alpha ^G$-node.
  - No proper initial segment of $(\bigcup G_0)(W \cap \kappa ^+)$ is an element of ${\mathrm{{ran}}(\dot{X}_{g,\alpha }^G)}$.

### Proof of Claim 4.18

In order to make use of Lemma 4.10, it will be more convenient to work with the transitive collapse of *W* instead of *W* itself. Let $H_W$ be the transitive collapse of *W*, and let ${\sigma }:{H_W}\longrightarrow {W \prec \mathrm{{H}}(\theta )}$ be the inverse of the collapsing map.

In the following, if *b* is a set, then we will write

$$\begin{aligned} \bar{b} ~ = ~ \sigma ^{{-}1} [b] ~ \subseteq ~ H_W. \end{aligned}$$

Note that $\bar{b}=\sigma ^{{-}1}(b)$ holds for all $b\in W$ and we will frequently use this abbreviation in the following arguments.

Since *g* is a $(W,{\mathbb {P}})$-generic filter, we know that $\bar{g}$ is a $\bar{{\mathbb {P}}}$-generic over $H_W$, and, in particular, it follows that $\bar{g}_\gamma$ is $\bar{{\mathbb {P}}}_\gamma$-generic over $H_W$ for all $\gamma \in W\cap \varepsilon$.

If we define

$$\begin{aligned} k ~ = ~ \{{q(\bar{\alpha })^{\bar{g}_\alpha }}~\vert ~{q\in \bar{g}_{\alpha +1}}\} ~ \subseteq ~ H_W[\bar{g}_\alpha ], \end{aligned}$$

then *k* is $\sigma ^{{-}1}(\dot{{\mathbb {Q}}}_\alpha )^{\bar{g}_\alpha }$-generic over $H_W[\bar{g}_\alpha ]$ with $H_W[\bar{g}_{\alpha +1}] = H_W[\bar{g}_\alpha ][k]$.

Set $\delta = (\kappa ^+)^{H_W}$, and note that $\delta = {\mathrm{{crit}}\left( {\sigma }\right) }$, because $\vert {W}\vert =\kappa \subseteq W$.

Since $\Phi _\alpha$ holds, we know that $p(f_0,g) \restriction \alpha$ is a condition in ${\mathbb {P}}_\alpha$ that extends every element of the $(W,{\mathbb {P}}_\alpha )$-generic filter $g_\alpha$. In particular, $p(f_0,g) \restriction \alpha$ it is a total master condition for *W*. By Lemma 2.2, every ${\mathbb {P}}_\alpha$-name for a function from $\kappa$ to the ordinals in *W* is forced by $p(f_0,g) \restriction \alpha$ to be evaluated to an element of *W*. It follows that

4 $$\begin{aligned} H_W \cap {}^\kappa \mathrm{{Ord}}~ = ~ H_W[\bar{g}_\alpha ] \cap {}^\kappa \mathrm{{Ord}}. \end{aligned}$$

Let $h_k$ be the union of the left coordinates of *k* and let $X_k$ be the union of the right coordinates of *k*.

By (4), we can apply Lemma 4.10 with the ground model $H_W$ and the outer model $H_W[\bar{g}_\alpha ]$ and derive the following statements:

- The function
  $$\begin{aligned} {h_k}:{T(\sigma ^{{-}1}(\dot{S}_\alpha )^{\bar{g}_\alpha })^{H_W[\bar{g}_\alpha ]}}\longrightarrow {\sigma ^{{-}1}(\dot{\mathcal {T}}(\dot{G}_0))^{\bar{g}_0}} \end{aligned}$$
  is order preserving and every element of its range is a $\sigma ^{{-}1}(\dot{S}_\alpha )^{\bar{g}_\alpha }$-node in $H_W[\bar{g}_\alpha ]$.
- No element of ${\mathrm{{ran}}(h_k)}$ extends an element of the range of the function
  $$\begin{aligned} {X_k}:{\delta }\longrightarrow {({}^{{<}\delta }\delta )^{H_W}}. \end{aligned}$$

Set $x=(h_k,X_k)$. In the following, we will show that $p(f_0,g) \restriction \alpha$ and *x* satisfy (3), and $p(f_0,g) \restriction \alpha$ forces the other statements of the claim to hold true.

Let *G* be ${\mathbb {P}}_\alpha$-generic over $\mathrm{{V}}$ with $p(f_0,g) \restriction \alpha \in G$. Work in *V*[*G*]. Since $\alpha \in W$ and $p(f_0,g) \restriction \alpha$ is a *W*-total master condition that, in particular, extends every element of $g_\alpha$, it follows that $W[G] \cap \mathrm{{V}}= W$, $G \cap W = g_\alpha$, and $\sigma$ can be canonically lifted to an elementary embedding

$$\begin{aligned} {\hat{\sigma }}:{H_W[\bar{g}_\alpha ]}\longrightarrow {W[G] \prec \mathrm{{H}}(\theta )[G]}. \end{aligned}$$

satisfying

$$\begin{aligned} \hat{\sigma }(\tau ^{\bar{g}_\alpha }) ~ = ~ \sigma (\tau )^G \end{aligned}$$

for every $\bar{{\mathbb {P}}}_\alpha$-name $\tau$ in $H_W$. Note that ${\mathrm{{ran}}(\hat{\sigma })} = W[G]$ holds.

Now, pick an element *q* of $\bar{g}_{\alpha +1}\subseteq H_W$. By the definition of $\hat{\sigma }$, we then have

$$\begin{aligned} \hat{\sigma }(q(\bar{\alpha })^{\bar{g}_\alpha }) ~ = ~ \sigma (q(\bar{\alpha }))^G ~ = ~ (\sigma (q)(\sigma (\bar{\alpha })))^G ~ = ~ (\sigma (q)(\alpha ))^G. \end{aligned}$$

Since $q\in \bar{g}_{\alpha +1}$ implies that $\sigma (q)\in g_{\alpha +1}$, we can now conclude that $\hat{\sigma }(q(\bar{\alpha })^{\bar{g}_\alpha })\in \dot{c}_{g,\alpha }^G$. These computations show that $\hat{\sigma }[k] \subseteq \dot{c}_{g,\alpha }^G$.

Next, fix a condition *p* in *g*. Since $g\cup \{\alpha \}\subseteq W={\mathrm{{ran}}(\sigma )}$, we then have $\bar{p}\restriction (\bar{\alpha }+1)\in \bar{g}_{\alpha +1}$ and $\bar{p}(\bar{\alpha })^{\bar{g}_\alpha }\in k$.

By the definition of $\hat{\sigma }$, we now know that

$$\begin{aligned} p(\alpha )^G ~ = ~ \sigma (\bar{p}(\bar{\alpha }))^G ~ = ~ \hat{\sigma }(\bar{p}(\bar{\alpha })^{\bar{g}_\alpha }) ~ \in ~ \hat{\sigma }[k]. \end{aligned}$$

This shows that $\dot{c}_{g,\alpha }^G\subseteq \hat{\sigma }[k]$ and, together with the above computations, we can conclude that

5 $$\begin{aligned} \hat{\sigma }[k] ~ = ~ \dot{c}_{g,\alpha }^G. \end{aligned}$$

By (5) and the elementarity of $\hat{\sigma }$, we also have $\hat{\sigma }[h_k]=\dot{h}_{g,\alpha }^G$ and $\hat{\sigma }[X_k]=\dot{X}_{g,\alpha }^G$.

Note that conditions in $\sigma ^{{-}1}(\dot{{\mathbb {Q}}}_\alpha )^{\bar{g}_\alpha }$ are elements of $\mathrm{{H}}(\delta )^{H_W[\bar{g}_\alpha ]}$ and hence *k* is a subset of $\mathrm{{H}}(\delta )^{H_W[\bar{g}_\alpha ]}$.

Since the critical point of $\hat{\sigma }$ is $\delta$, it follows that *k*, $h_k$ and $X_k$ are all pointwise fixed by $\hat{\sigma }$. In particular, we have

6 $$\begin{aligned} x ~ = ~ (h_k,X_k) ~ = ~ (\dot{h}_{g,\alpha }^G,\dot{X}_{g,\alpha }^G). \end{aligned}$$

Since $p(f_0,g)(\alpha )$ is, by definition, the ${\mathbb {P}}_\alpha$-name $\mathsf {pair}_{{\mathbb {P}}_\alpha }(\dot{h}_{g,\alpha },\dot{X}_{g,\alpha })$, this completes the proof of (3).

Part (1) of the claim follows by the properties of $(h_k,X_k)$ over $H_W[\bar{g}_\alpha ]$ discussed above, together with the equality (6), elementarity of $\hat{\sigma }$, and the fact that $\hat{\sigma }$ fixes bounded subsets of $\delta$ that lie in $H_W[\bar{g}_\alpha ]$. For example, to verify requirement (4.8(a)iv) of Definition 4.8, suppose *t* is in the range of $h_k$. Then *t* is in the range of the left coordinate of some condition in $k \subseteq \hat{\sigma }^{{-}1}({\mathbb {P}}(\dot{S}_\alpha ^G,G_0)^{\mathrm{{V}}[G]})$, and hence *t* is an $\hat{\sigma }^{{-}1}(\dot{S}_\alpha ^G)$-node in $H_W[\bar{g}_\alpha ]$. By elementarity of $\hat{\sigma }$ and the fact that $\hat{\sigma }$ fixes *y*, it follows that *t* is an $\dot{S}_\alpha ^G$-node in $\mathrm{{V}}[G]$.

The remaining requirements of Definition 4.8, except for requirement (3(a)vi), are easily verified for the pair displayed in (6) in a similar manner.

Now, we prove that $p(f_0,g) \restriction \alpha$ forces the statements in part (2) of the claim. Recall *G* is an arbitrary ${\mathbb {P}}_\alpha$-generic filter over $\mathrm{{V}}$ with $p(f_0,g) \restriction \alpha \in G$. Assume that the ordered pair (6) is **not** a condition in ${\mathbb {P}}(\dot{S}_\alpha ^G,G_0)$ in $\mathrm{{V}}[G_i]$. In the following, we show that the statements (2a), (2b), and (2c) of the claim hold true in $\mathrm{{V}}[G]$. By part (1) of the claim, it must be requirement (3(a)vi) of Definition 4.8 that fails. In particular, there is an increasing sequence $\vec {y}=\langle {y_\zeta }~\vert ~{\zeta < \kappa }\rangle$ of nodes in the range of $h_k$ in $\mathrm{{V}}[G]$ such that the function $f_{\bar{y}}=\bigcup _{\zeta < \kappa } y_\zeta$ is not an element of $\mathcal {T}(G_0)$. Since the elements of the range of $h_k$ are $\kappa$-sized objects in $H_W[\bar{g}_\alpha ]$ and hence in $H_W$ by (4), this implies that the domain of $f_{\vec {y}}$ is at most $\delta = (\kappa ^+)^{H_W}$. In summary, there is some ordinal $\gamma \in (S^{\kappa ^+}_\kappa )^\mathrm{{V}}$ such that

7 $$\begin{aligned} V[G] \models {\text {``}{{\gamma ~ \le ~ \delta ~ = ~ (\kappa ^+)^{H_W} ~ and ~ f_{\vec {y}}\restriction \gamma ~ = ~ (\textstyle {\bigcup G_0})(\gamma )}}\text {''}}. \end{aligned}$$

By part (4) of Lemma 4.10 – viewing $H_W$ as the ground model $\mathrm{{V}}$, $H_W[\bar{g}_\alpha ]$ as the outer model $\mathrm{{V}}_1$, *k* as the generic filter *K*, and *V*[*G*] as the outer model *M* from the statement of that lemma – the ordinal $\gamma$ cannot be strictly smaller than $\delta$, and hence we can conclude that $\gamma = \delta$. But this implies that ${\mathrm{{cof}}(W\cap \kappa ^+)}^\mathrm{{V}}={\mathrm{{cof}}(\delta )}^\mathrm{{V}}={\mathrm{{cof}}(\gamma )}^\mathrm{{V}}=\kappa$, proving part (2a) of the claim.

In summary, we have shown that $W \cap \kappa ^+ = {\mathrm{{dom}}(f_{\vec {y}})}$ and

8 $$\begin{aligned} f_{\vec {y}} ~ = ~ (\textstyle {\bigcup G_0})(W \cap \kappa ^+). \end{aligned}$$

Since $f_{\vec {y}}$ is a union of functions in the range of $h_k$, and (4) together with the fact that the critical point of $\sigma$ is $\delta$ imply that $h_k \subseteq W$, every proper initial segment of $f_{\vec {y}}$ is an element of *W*. Furthermore, every proper initial segment of $f_{\vec {y}}$ is extended by some $y \in {\mathrm{{ran}}(h_k)}$, which is an $\dot{S}_\alpha ^G$-node in $\mathrm{{V}}[G]$ by part (1) of the claim. Hence, every proper initial segment of $f_{\vec {y}}$ is an $\dot{S}_\alpha ^G$-node in $\mathrm{{V}}[G]$, and is an element of *W*. Together with (8), this proves part (2b) of the claim. Finally, to prove part (2c) of the claim, suppose $\gamma \in {\mathrm{{dom}}(X_k)}$, define $\eta = {\mathrm{{dom}}(X_k(\gamma ))}$ and assume, towards a contradiction, that $f_{\vec {y}} \restriction \eta = \bar{X}(\alpha )$. Note that $\eta < \delta$, because $X_k \subseteq H_W[\bar{g}_\alpha ]$. In particular, we have $y_\zeta \restriction \eta = X_k(\alpha )$ for some $\zeta <\kappa$. But this contradicts the fact from part (1) that nothing in the range of $h_k$ extends any function from the range of $X_k$. $\square$

It remains to prove part (2c) of the lemma, which will essentially follow from part (2) of Claim 4.18, though we first must dispense with a technicality. Recall that $\Phi _{\alpha +1}$ fails, but $\Phi _\alpha$ holds. Next, we observe that the failure of $\Phi _{\alpha +1}$ is due to the function $p(f_0,g) \restriction (\alpha +1)$ not being a condition at all (rather than being a condition but failing to extend $g_{\alpha +1}$, or being a condition but failing to be flat):

### Claim 4.19

Some condition in ${\mathbb {P}}_\alpha$ below $p(f_0,g) \restriction \alpha$ forces that

9 $$\begin{aligned} p(f_0,g)(\alpha ) ~ = ~ \mathsf {pair}_{{\mathbb {P}}_\alpha }(\dot{h}_{g,\alpha },\dot{X}_{g,\alpha }) \end{aligned}$$

is **not** a condition in $\dot{{\mathbb {Q}}}_\alpha$.

### Proof of Claim 4.19

Assume not, i.e. suppose that $p(f_0,g) \restriction \alpha$ forces that the pair in (9) to be a condition in $\dot{{\mathbb {Q}}}_\alpha$. Since the components of the pair in (9) are given by the union of the left and right coordinates of $\dot{c}_{g,\alpha }$, the fact that the ordering of $\dot{{\mathbb {Q}}}_\alpha$ is given reversed inclusion now implies that the condition $p(f_0,g) \restriction \alpha$ forces $p(f_0,g)(\alpha )$ to be stronger than every condition in $\dot{c}_{g,\alpha }$. Since the validity of $\Phi _\alpha$ implies that $p(f_0,g) \restriction \alpha$ is stronger than every condition in $g_\alpha$, it follows that

$$\begin{aligned} p(f_0,g) \restriction (\alpha +1) ~ = ~ (p(f_0,g) \restriction \alpha )^\frown (\alpha ,\mathsf {pair}_{{\mathbb {P}}_\alpha } (\dot{h}_{g,\alpha },\dot{X}_{g,\alpha })) \end{aligned}$$

is stronger than every condition in $g_{\alpha +1}$.

Furthermore, by Claim 4.18, there is an $x_\alpha \in \mathrm{{V}}$ such that

$$\begin{aligned} p(f_0,g) \restriction \alpha ~ \Vdash _{{\mathbb {P}}_\alpha } ~ {\text {``}{\check{x}_\alpha = p(f_0,g)(\alpha )}\text {''}}. \end{aligned}$$

Since $\Phi _\alpha$ holds, we know that $p(f_0,g) \restriction \alpha$ is flat. Let $\langle {x_\ell }~\vert ~{\ell \in W \cap \alpha }\rangle$ witness its flatness. Then the sequence $\langle {x_\ell }~\vert ~{\ell \in W \cap (\alpha +1)}\rangle$ witnesses the flatness of $p(f_0,g) \restriction (\alpha +1)$. In summary, $p(f_0,g) \restriction (\alpha +1)$ is a flat condition below every member of $g_{\alpha +1}$, contradicting the fact that $\Phi _{\alpha +1}$ fails. $\square$

Part (2c) of the lemma now follows immediately from Claim 4.19, and part (2) of Claim 4.18. $\square$

The above results now allow us to prove the following key lemma.

### Lemma 4.20

The poset ${\mathbb {P}}$ is $\kappa ^+$-Jensen complete, and the flat conditions are dense in ${\mathbb {P}}$.

Before we prove this result, we make a couple of remarks.

### Remark 4.21

In [13,  Claim 3.13], a weaker version of Lemma 4.20, stating that ${\mathbb {P}}$ is $\kappa$-*proper*, was proven. This concept was defined in [13,  Definition 3.4] and only makes sense under the assumption that $\kappa ^{{<}\kappa } = \kappa$. It, in particular, implies that the given poset is ${<}\kappa ^+$-distributive.

In the non-$\mathrm{{GCH}}$ setting, in particular, when we do not assume $\kappa ^{{<}\kappa } = \kappa$, perhaps the most natural analogue of $\kappa$-properness is our notion of $\text {IA}_\kappa$-proper (Defininition 2.8). In fact, changing just a few words in the proof of [13,  Claim 3.13] would suffice to prove that (even without $\mathrm{{GCH}}$) the poset ${\mathbb {P}}$ is proper for $\text {IA}_\kappa$ and is ${<}\kappa ^+$-distributive. However, that conclusion does not suffice for applications in our main theorems, since, for example, $\text {IA}_{\omega _1}$-properness, even together with ${<}\omega _2$-strategic closure, does not guarantee preservation of the *Proper Forcing Axiom*.^18^

We seem to need the stronger property of $\kappa ^+$-Jensen completeness (i.e. ${<}\kappa ^+$-directed closure), which we prove in Lemma 4.20. This requires some reorganization and strengthening of the argument of [13,  Claim 3.13], but the main ideas of the proof of Lemma 4.20 are very similar to the proof of [13,  Claim 3.13].

### Remark 4.22

Iterations using ${<}\kappa ^+$-support, where each iterand is ${<}\kappa ^+$-directed closed, are themselves ${<}\kappa ^+$-directed closed. However, this fact seems to *not* be applicable to the iteration ${\mathbb {P}}_\varepsilon$ constructed in Definition 4.12. That is, it is not clear if, say, the first non-trivial poset used of the form ${\mathbb {P}}(S,G_0)$ is equivalent to a ${<}\kappa ^+$-directed closed from the point of view of $V[G_0]$ (and we suspect it is not, in general). The key to Lemma 4.20 (and to the analogous, but weaker [13,  Claim 3.13]) is the flexibility in having $G_0$ not be decided yet.

### Proof of Lemma 4.20

First, we check $\kappa ^+$-Jensen completeness. Since each iterand is ${<}\kappa$-closed and the iteration uses $\kappa$-sized supports, the entire iteration is ${<}\kappa$-closed. So by Corollary 2, to show that ${\mathbb {P}}$ is $\kappa ^+$-Jensen complete, it suffices to show that whenever

- $W \prec (\mathrm{{H}}(\theta ),\in ,{\mathbb {P}})$ with $\vert {W}\vert =\kappa$ and $W \cap \kappa ^+ \in \kappa ^+$, and
- $g \subseteq W \cap {\mathbb {P}}$ is a $(W,{\mathbb {P}})$-generic filter,

then *g* has a lower bound in ${\mathbb {P}}$. So fix such a filter *g* for the remainder of the proof. Given $\alpha \in W \cap [0,\varepsilon ]$, define $g_\alpha$ as in Definition 4.15. Set $\delta = W \cap \kappa ^+$. We consider two cases:

**Case 1:** ${\mathrm{{cof}}(\delta )}< \kappa$. Set $f_0=\bigcup g_0$, and consider the function $p(f_0,g)$ from Definition 4.15. We claim that $p(f_0,g)$ is flat condition and lies below all members of *g*.

Assume not. Then, by Lemma 4.16, there is an $\alpha \in W \cap [1,\varepsilon )$ such that $p(f_0,g) \restriction \alpha$ is a condition below all conditions in $g_\alpha$, and there is some $q_\alpha \le _{{\mathbb {P}}_\alpha } p(f_0,g) \restriction \alpha$ in ${\mathbb {P}}_\alpha$ that forces all the statements in part (2c) of Lemma 4.16 to hold. In particular, by part (2(c)i), we know that ${\mathrm{{cof}}(\delta )} ={\mathrm{{cof}}(W \cap \kappa ^+)}=\kappa$, contrary to our case.

**Case 2**: ${\mathrm{{cof}}(\delta )}= \kappa$. Since $\vert {W}\vert =\kappa < \delta$, we can fix ${t}:{\delta }\longrightarrow {\delta }$ such that $t \restriction \kappa$ is not an element of *W*. Define

$$\begin{aligned} f_0 ~ = ~ (\textstyle {\bigcup g_0}) \cup \{(\delta ,t)\}. \end{aligned}$$

Given $\gamma \in \delta \cap S^{\kappa ^+}_\kappa$, we have $t \restriction \gamma \notin W$ and $(\bigcup g_0)(\gamma ) \in W$. In particular, we have $t \restriction \gamma \ne (\bigcup g_0)(\gamma )$ for all $\gamma \in \delta \cap S^{\kappa ^+}_\kappa$. Since ${\mathrm{{cof}}(\delta )} = \kappa$, this shows that $f_0$ is a condition in ${\mathbb {Q}}_0$ that extends $\bigcup g_0$.

Let $p(f_0,g)$ be the function defined in Definition 4.15. We claim that $p(f_0,g)$ is a flat condition that lies below every element of *g*. Assume not. Then, by Lemma 4.16, there is an $\alpha \in W \cap [1,\varepsilon )$ such that $p(f_0,g) \restriction \alpha$ is a condition in ${\mathbb {P}}_\alpha$, and that, by part (2(c)ii) of that lemma, there is some condition $q \le _{{\mathbb {P}}_\alpha } p(f_0,g) \restriction \alpha$ such that *q* forces that every proper initial segment of $(\bigcup \dot{G}_0)(\check{\delta })$ is an element of *W*. But the 0th component of $p(f_0,g) \restriction \alpha$, and hence of *q*, extends the function $f_0$, and therefore

$$\begin{aligned} q(0)\Vdash _{{\mathbb {P}}_0}{\text {``}{(\textstyle {\bigcup \dot{G}_0})(\check{\delta }) = \check{t}}\text {''}}. \end{aligned}$$

In particular, every proper initial segment of *t* is an element of *W*, contrary to our choice of *t*.

This completes the proof of $\kappa ^+$-Jensen completeness. To see that the flat conditions are dense in ${\mathbb {P}}$, let $p_0$ be any condition in ${\mathbb {P}}$. Fix $W \prec (\mathrm{{H}}(\theta ),\in ,{\mathbb {P}},p_0)$ such that $\vert {W}\vert =\kappa \subseteq W$ and $W \in \text {IA}_\kappa$. By Lemma 2.7 and the ${<}\kappa$-closure of ${\mathbb {P}}$, there exists a $(W,{\mathbb {P}})$-generic filter *g* such that $p_0 \in g$. Note that $W \in \text {IA}_\kappa$ implies that ${\mathrm{{cof}}(W \cap \kappa ^+)} = \kappa$. This shows that we can repeat the argument from the above Case 2, define $f_0$ as above and conclude that the function $p(f_0,g)$ is a flat condition that is below every member of *g* and therefore also below $p_0$. $\square$

Lemma 4.20 and Corollary 2 now immediately yield the following corollary:

### Corollary 3

The poset ${\mathbb {P}}$ is forcing equivalent to a ${<}\kappa ^+$-directed closed forcing. In particular, it adds no new sets of size $\kappa$, and, in the case $\kappa = \omega _1$, it preserves all standard forcing axioms, such as $\mathrm{{MM}}^{++}$. $\square$

Remember that the order *o*(*p*) of a condition in a poset of the form ${\mathbb {P}}(S,G_0)$ was defined in part (4) of Definition 4.8.

### Lemma 4.23

If *p* is a flat condition in ${\mathbb {P}}$, then there exists $\beta < \kappa ^+$ with the property that

$$\begin{aligned} p\restriction \alpha \Vdash _{{\mathbb {P}}_\alpha }{\text {``}{o(p(\alpha ))\le \check{\beta }}\text {''}} \end{aligned}$$

holds for all $1\le \alpha \in {\mathrm{{sprt}}(p)}$.

### Proof

Let $\langle {x_\alpha }~\vert ~{\alpha \in {\mathrm{{sprt}}(p)}}\rangle$ be a sequence witnessing the flatness of *p*. For each $\alpha \in {\mathrm{{sprt}}(p)}$, pick $\beta _\alpha < \kappa ^+$ such that $\beta _\alpha$ is not in the transitive closure of $x_\alpha$. Since $\vert {{\mathrm{{sprt}}(p)}}\vert \le \kappa$, we know that

$$\begin{aligned} \beta ~ = ~ \sup \{{\beta _\alpha }~\vert ~{\alpha \in {\mathrm{{sprt}}(p)}}\} ~ < ~ \kappa ^+ \end{aligned}$$

has the desired properties. $\square$

Our next task is to prove that tails of the iteration behave nicely. But first we need *tail* versions of Definition 4.15 and Lemma 4.16. Note that in Definition 4.24 below, since $\alpha _0 \ge 1$, the entire filter $G_0$ has already been determined. So unlike Definition 4.15, the candidate for a condition below *g* will not involve any $f_0$.

### Definition 4.24

Suppose that $\alpha _0 \in [1,\varepsilon )$ and $G_{\alpha _0}$ is ${\mathbb {P}}_{\alpha _0}$-generic over $\mathrm{{V}}$. Working in $\mathrm{{V}}[G_{\alpha _0}]$, suppose that $W \prec (\mathrm{{H}}(\theta )[G_{\alpha _0}], \in , {\mathbb {P}}/G_{\alpha _0})$ with $\vert {W}\vert =\kappa \subseteq W$, and $g \subseteq W \cap {\mathbb {P}}/G_{\alpha _0}$ is a $(W,{\mathbb {P}}/G_{\alpha _0})$-generic filter. For each $\alpha \in W \cap [\alpha _0,\varepsilon )$, define ${\mathbb {P}}_\alpha /G_{\alpha _0}$-names $\dot{c}_{g,\alpha }$, $\dot{h}_{g,\alpha }$ and $\dot{X}_{g,\alpha }$ analogously to Definition 4.15, and define a function *p*(*g*) with domain $W \cap [\alpha _0,\varepsilon )$ by setting

$$\begin{aligned} p(g) ~ = ~ \langle {\mathsf {pair}_{{\mathbb {P}}_\alpha /{G_{\alpha _0}}}(\dot{h}_{g,\alpha },\dot{X}_{g,\alpha })}~\vert ~{i \in W \cap [\alpha _0,\varepsilon )}\rangle . \end{aligned}$$

We now also have a *tail* variant of Lemma 4.16:

### Lemma 4.25

Suppose $\alpha _0 \in [1,\varepsilon )$ and $G_{\alpha _0}$ is ${\mathbb {P}}_{\alpha _0}$-generic over $\mathrm{{V}}$. Work in $V[G_{i_0}]$ and suppose *W* and *g* are as in Definition 4.24. Given $\alpha \in [\alpha _0,\varepsilon ]$, set

$$\begin{aligned} g_\alpha ~ = ~ \{{p\restriction \alpha }~\vert ~{p\in g}\}. \end{aligned}$$

Then one of the following statements holds:

1. *p*(*g*) is a flat condition in ${\mathbb {P}}/G_{\alpha _0}$ that extends every element of *g*.
2. There is an $\alpha \in W \cap [\alpha _0,\varepsilon )$ such that the following statements hold:
  - $p(g) \restriction \alpha$ is a flat condition in ${\mathbb {P}}_\alpha /G_{\alpha _0}$ that is stronger than every element of $g_\alpha$.
  - If $\tau \in W$ is a ${\mathbb {P}}_\alpha /G_{\alpha _0}$-name for a function from $\kappa$ to the ordinals, then
    $$\begin{aligned} p(g) \restriction \alpha \Vdash _{{\mathbb {P}}_\alpha /G_{\alpha _0}}{\text {``}{\tau \in \check{W}}\text {''}}. \end{aligned}$$
  - There is a condition *q* in ${\mathbb {P}}_\alpha /G_{\alpha _0}$ below $p(g)\restriction \alpha$ with the property that the following statements hold true in $\mathrm{{V}}[G_{\alpha _0},G]$, whenever *G* is ${\mathbb {P}}_\alpha /G_{\alpha _0}$-generic over $\mathrm{{V}}[G_{\alpha _0}]$ with $q\in G$:
    (i) $${\mathrm{{cof}}(W \cap \kappa ^+)} = \kappa $$.
    (ii) Every proper initial segment of $(\bigcup G_0)(W \cap \kappa ^+)$ is an element of *W*, and is an $\dot{S}_\alpha ^G$-node.
    (iii) No proper initial segment of $(\bigcup G_0)(W \cap \kappa ^+)$ is an element of ${\mathrm{{ran}}(\dot{X}_{g,\alpha }^G)}$.

### Proof

The proof is almost identical to the proof of Lemma 4.16, except we work in $\mathrm{{V}}[G_{\alpha _0}]$ instead of $\mathrm{{V}}$. We leave the details to the reader. $\square$

### Lemma 4.26

If $\alpha < \varepsilon$ and *G* is ${\mathbb {P}}_\alpha$-generic over $\mathrm{{V}}$, then the tail of the iteration ${\mathbb {P}}/G$ is ${<}\kappa$-closed in $\mathrm{{V}}[G]$.

### Proof

Let $\langle {q_\xi }~\vert ~{\xi <\mu }\rangle$ be a descending sequence with $\mu <\kappa$ in ${\mathbb {P}}/G$ in $\mathrm{{V}}[G]$. Since ${\mathbb {P}}/G\subseteq {\mathbb {P}}$ and Lemma 4.20 shows that ${\mathbb {P}}_\alpha$ is ${<}\kappa$-closed in $\mathrm{{V}}$, this sequence is an element of $\mathrm{{V}}$. Let $\dot{G}$ denote the canonical ${\mathbb {P}}_\alpha$-name for the generic filter in $\mathrm{{V}}$. Fix a condition *p* in *G* such that

$$\begin{aligned} p\Vdash _{{\mathbb {P}}_\alpha }{\text {``}{{Every\, condition\, in\, \dot{G}\, is \, compatible\, with\, \check{q}_\xi \, in\, \check{{\mathbb {P}}}}}\text {''}} \end{aligned}$$

holds in $\mathrm{{V}}$ for all $\xi <\mu$. Work in $\mathrm{{V}}$. Given $\xi <\mu$, a standard density argument now shows that

$$\begin{aligned} p\Vdash _{{\mathbb {P}}_\alpha }{\text {``}{\check{q}_\xi \restriction \check{\alpha }\in \dot{G}}\text {''}} \end{aligned}$$

and the separativity of ${\mathbb {P}}_\alpha$ allows us to conclude that $p\le _{{\mathbb {P}}_\alpha }q\restriction \alpha$ holds.

Fix a condition *r* below *p* in ${\mathbb {P}}_\alpha$ and set

$$\begin{aligned} r_\xi ~ = ~ r^\frown (q_\xi \restriction [\alpha , \varepsilon )) \end{aligned}$$

for all $\xi <\mu$. Then $\langle {r_\xi }~\vert ~{\xi <\mu }\rangle$ is a descending sequence of conditions in ${\mathbb {P}}$, and, by the proof of Lemma 4.20, this sequence has a lower bound $r_\mu$ in ${\mathbb {P}}$. Then $r_\mu \restriction \alpha \le _{{\mathbb {P}}_\alpha }r$ and $r_\mu \le _{\mathbb {P}}q_\xi$ for all $\xi <\mu$.

By genericity, we can now find a condition *q* in ${\mathbb {P}}$ with the property that $q\restriction \alpha \in G$ and $q\le _{\mathbb {P}}q_\xi$ for all $\xi <\mu$. But then we can conclude that *q* is a condition in ${\mathbb {P}}/G$ in $\mathrm{{V}}[G]$ with $q\le _{{\mathbb {P}}/G}q_\xi$ for all $\xi <\mu$. $\square$

The proof of the following lemma is similar to the proof of [13,  Claim 3.14], but there are some subtle differences since we do not assume that $\kappa ^{{<}\kappa } = \kappa$. Roughly, we replace their use of $\kappa$-*properness* with $\text {IA}_\kappa$-properness (Definition 2.8) and verify that the argument still goes through.

### Lemma 4.27

If $\alpha _0< \varepsilon$ and *G* is ${\mathbb {P}}_{\alpha _0}$-generic over $\mathrm{{V}}$, then the tail of the iteration ${\mathbb {P}}/G$ is $\text {IA}_\kappa$-totally proper in $\mathrm{{V}}[G]$.

### Proof

For $\alpha _0 = 0$, the statement of the lemma follows immediately from Lemmas 2.7 and 4.20. Therefore, we from now on assume that $1\le \alpha _0<\varepsilon$.

Let $G_{\alpha _0}$ be ${\mathbb {P}}_{\alpha _0}$-generic over $\mathrm{{V}}$ and work in $\mathrm{{V}}[G_{\alpha _0}]$. Let $\theta$ be a sufficiently large regular cardinal, let $\lhd$ be a well-ordering of $\mathrm{{H}}(\theta )=\mathrm{{H}}(\theta )^\mathrm{{V}}[G_{\alpha _0}]$, let

$$\begin{aligned} W\prec (\mathrm{{H}}(\theta ),\in ,\alpha _0,{\mathbb {P}}/G_{\alpha _0},\lhd ) \end{aligned}$$

with $W\in \text {IA}_\kappa$, and let $p_0$ be a condition in $W \cap ({\mathbb {P}}/G_{\alpha _0})$. In the following, we will find a $(W,{\mathbb {P}}/G_{\alpha _0})$-total master condition below $p_0$. Define

$$\begin{aligned} t ~ = ~ (\textstyle {\bigcup G_0})(W \cap \kappa ^+), \end{aligned}$$

which is well-defined because $W \in \text {IA}_\kappa$ implies that ${\mathrm{{cof}}(W \cap \kappa ^+)}=\kappa$.

**Case 1:** *There exists* $\zeta \in W \cap \kappa ^+$ *with* $t \restriction \zeta \notin W$. Since Lemma 4.26 implies that ${\mathbb {P}}/G_{\alpha _0}$ is ${<}\kappa$-closed, we can apply Lemma 2.7 to find a $(W,{\mathbb {P}}/G_{\alpha _0})$-generic filter *g* that includes $p_0$. Let *p*(*g*) be the function defined in Definition 4.24 and assume, towards a contradiction, that *p*(*g*) is not a condition in ${\mathbb {P}}/G_{\alpha _0}$ that is stronger than every element of *g*. Then, by part (2(c)ii) of Lemma 4.25, there is an $\alpha \in W \cap [\alpha _0,\varepsilon )$ and some condition in ${\mathbb {P}}_\alpha /G_{\alpha _0}$ below $p(g)\restriction \alpha$ forcing that every proper initial segment of $(\bigcup G_0)(W \cap \kappa ^+)$ is an element of *W*. But this implies that every proper initial segment of *t* is an element of *W*, contrary to our case. This allows us to conclude that *p*(*g*) is a $(W,{\mathbb {P}}/G_{\alpha _0})$-total master condition below $p_0$.

**Case 2:** *If* $\zeta \in W\cap \kappa ^+$, *then* $t\restriction \zeta \in W$. Since $W \in \text {IA}_\kappa$, there is a sequence

$$\begin{aligned} \vec {D} ~ = ~ \langle {D_\xi }~\vert ~{\xi < \kappa }\rangle \end{aligned}$$

listing all open dense subsets of ${\mathbb {P}}/G_{\alpha _0}$ that are elements of *W*, and such that every proper initial segment of $\vec {D}$ is an element of *W*. Recursively define a descending sequence $\vec {p}=\langle {p_\xi }~\vert ~{\xi < \kappa }\rangle$ of conditions in ${\mathbb {P}}/G_{\alpha _0}$ as follows:

- Given $\xi <\kappa$, let $p^*_\xi$ be the $\lhd$-least flat condition in ${\mathbb {P}}/G_{\alpha _0}$ below $p_\xi$ that is an element of $D_\xi$. Such a condition exists by Lemma 4.20 and the open density of $D_\xi$. By Lemma 4.23, there exists $\beta < \kappa ^+$ with
  $$\begin{aligned} p^*_\xi \restriction \alpha \Vdash _{{\mathbb {P}}_\alpha }{\text {``}{o(p^*_\xi (\alpha ))\le \check{\beta }}\text {''}} \end{aligned}$$
  for all $1\le \alpha \in {\mathrm{{sprt}}(p^*_\xi )}$. Given $\alpha \in [\alpha _0,\varepsilon )$, let $\dot{f}_\alpha$ and $\dot{Y}_\alpha$ denote the canonical ${\mathbb {P}}_\alpha /G_{\alpha _0}$-names with the property that
  $$\begin{aligned} p^*_\xi (\alpha )^G ~ = ~ (\dot{f}_\alpha ^G,\dot{Y}_\alpha ^G) \end{aligned}$$
  holds whenever *G* is $({\mathbb {P}}_\alpha /G_{\alpha _0})$-generic over $\mathrm{{V}}[G_{\alpha _0}]$ with $p^*_\xi \restriction \alpha \in G$. Note that, by the choice of $\beta$, for each $\alpha \in {\mathrm{{sprt}}(p^*_\xi )}$, the condition $p^*_\xi \restriction \alpha$ forces that $\beta$ is larger than all domains of elements of the range of $\dot{f}_\alpha$, and larger than all elements in the domain of $\dot{Y}_\alpha$. In particular, if $\alpha _0\le \alpha \in {\mathrm{{sprt}}(p^*_\xi )}$ and *G* is $({\mathbb {P}}_\alpha /G_{\alpha _0})$-generic over $\mathrm{{V}}[G_{\alpha _0}]$ with $p^*_\xi \restriction \alpha \in G$, then
  10 $$\begin{aligned} (\dot{f}_\alpha ^G, ~ \dot{Y}_\alpha ^G ~ \cup ~ \{(\beta +1, ~ t\restriction (\beta + 1))\}) \end{aligned}$$
  is a condition in $\dot{{\mathbb {Q}}}_\alpha ^G$ below $p^*_\xi (\alpha )^G$.^19^ This shows that there is a condition $p_{\xi +1}$ below $p^*_\xi$ in ${\mathbb {P}}$ with ${\mathrm{{sprt}}(p_{\xi +1})}={\mathrm{{sprt}}(p^*_\xi )}$ and the property that whenever $\alpha _0\le \alpha \in {\mathrm{{sprt}}(p_{\xi +1})}$ and *G* is $({\mathbb {P}}_\alpha /G_{\alpha _0})$-generic over $\mathrm{{V}}[G_{\alpha _0}]$ with $p^*_\xi \restriction \alpha \in G$, then $p_{\xi +1}(\alpha )^G$ is equal to the condition in (10).
  Now, assume that $p_\xi$ is an element of *W*. Then $p^*_\xi$ is obviously definable in $(\mathrm{{H}}(\theta )[G_{\alpha _0}], \in ,{\mathbb {P}}, G_{\alpha _0},\lhd )$ using the parameters $p_\xi$ and $D_\xi$, which are both contained in *W*. Since $p^*_{\xi } \in W$, then $\beta$ can also be taken to be an element of *W*. Finally, the condition $p_{\xi +1}$ is definable from $p^*_\xi$, $\beta$, and $t \restriction (\beta + 1)$, all of which are elements of *W* because of the case we are in. These arguments show that $p_\xi \in W$ implies that $p_{\xi + 1}\in W$.
- If $\xi < \kappa$ is a limit ordinal, then we define $p_\xi$ be the $\lhd$-least lower bound of the sequence $\langle {p_\zeta }~\vert ~{\zeta <\xi }\rangle$ in ${\mathbb {P}}$.^20^

Note that every proper initial segment of $\vec {p}$ is an element of *W*, because *W* contains all proper initial segments of *t* and each proper initial segment of $\vec {p}$ is definable in the structure $(\mathrm{{H}}(\theta )[G_{\alpha _0}],\in ,\lhd ,{\mathbb {P}})$ using the parameter $p_0$ and some sufficiently long^21^ proper initial segment of *t*. Hence, not only is each $p_{\xi +1}$ an element of $D_\xi$, but is in fact an element of $D_\xi \cap W$. In particular, the set $\{{p_\xi }~\vert ~{\xi < \kappa }\}$ generates a $(W,{\mathbb {P}}/G_{\alpha _0})$-generic filter. Let *g* denote this filter, and let *p*(*g*) be the function defined in Definition 4.24.

Now, assume, towards a contradiction, that *p*(*g*) is not a condition below every member of *g*. Then by part (2(c)iii) of Lemma 4.25, there is an $\alpha \in W \cap [\alpha _0,\varepsilon )$ and a condition *q* in ${\mathbb {P}}_\alpha /G_{\alpha _0}$ below $p(g)\restriction \alpha$ with the property that whenever *G* is $({\mathbb {P}}_\alpha /G_{\alpha _0})$-generic over $\mathrm{{V}}[G_{\alpha _0}]$ with $q\in G$, then no proper initial segment of $t=(\bigcup G_0)(W \cap \kappa ^+)$ is an element of ${\mathrm{{ran}}(\dot{X}_{g,\alpha }^G)}$. Since $\alpha \in W$ and the set $\{{p_\xi }~\vert ~{\xi < \kappa }\}$ generates the $(W,{\mathbb {P}}/G_{\alpha _0})$-generic filter *g*, we can find $\xi _\alpha <\kappa$ with the property that $\alpha \in {\mathrm{{sprt}}(p_{\xi _\alpha + 1})}$. Then $q\le _{}p_{\xi _\alpha +1}\restriction \alpha$. Let *G* be $({\mathbb {P}}_\alpha /G_{\alpha _0})$-generic over $V[G_{\alpha _0}]$ with $q \in G$. Work in $V[G_{\alpha _0},G]$. Since $p_{\xi _\alpha + 1} \in g$, we know that $p_{\xi _\alpha + 1}(\alpha )^G \in \dot{c}_{g,\alpha }^G$ and hence $\dot{X}_{g,\alpha }^G$ extends the right coordinate of $p_{\xi _\alpha + 1}(\alpha )^G$. By construction, the range of the right coordinate of $p_{\xi _\alpha + 1}(\alpha )^G$ contains a proper initial segment of *t*, contradicting the properties of $\alpha$ and *q*.

Again, we can conclude that *p*(*g*) is a $(W,{\mathbb {P}}/G_{\alpha _0})$-total master condition below the condition $p_0$. $\square$

### Corollary 4

Let *G* be ${\mathbb {P}}$-generic over $\mathrm{{V}}$, let $\alpha \in (0,\varepsilon )$, let $G_\alpha$ be the filter on ${\mathbb {P}}_\alpha$ induced by *G*, and let $S=\dot{S}_\alpha ^{G_\alpha }$. Then $(S^{\kappa ^+}_\kappa \setminus S)^{\mathrm{{V}}[G_\alpha ]}$ is stationary in *V*[*G*].

### Proof

First, if $S=\emptyset$, then the conclusion of the lemma holds trivially, because Corollary 3 implies that $(S^{\kappa ^+}_\kappa )^{\mathrm{{V}}[G_\alpha ]}=(S^{\kappa ^+}_\kappa )^{\mathrm{{V}}[G]}$. In the other case, we know that $S^{\kappa ^+}_\kappa \setminus S$ contains a stationary set in $I[\kappa ^+]$ in $\mathrm{{V}}[G_\alpha ]$, and hence a combination of Lemma 2.12, Corollary 3 and Lemma 4.27 ensures that $S^{\kappa ^+}_\kappa \setminus S$ remains stationary in $\mathrm{{V}}[G]$. $\square$

### Lemma 4.28

If *G* is ${\mathbb {P}}$-generic over $\mathrm{{V}}$, then the tree $\mathcal {T}(G_0)$ has no cofinal branches in $\mathrm{{V}}[G]$.

Our proof of this lemma is similar to the proof of [13,  Claim 3.15], but we must make the following changes:

- Whereas the proof of [13,  Claim 3.15] makes use of ${<}\kappa$-closed elementary submodels of size $\kappa$ (whose existence requires the assumption $\kappa ^{{<}\kappa } = \kappa$), we instead use elementary submodels in $\text {IA}_\kappa$.
- We use Corollary 4 to ensure that the complement of each $\dot{S}_\alpha$ is stationary in the final model (this is used to get the right analogue of statement (8) on page 1691 of [13]).

### Proof of Lemma 4.28

Let $\dot{b}$ be a ${\mathbb {P}}$-name for a function from $\kappa ^+$ to $\kappa ^+$. Assume, towards a contradiction, that there is a condition *p* in ${\mathbb {P}}$ that forces $\dot{b}$ to be a cofinal branch through $\mathcal {T}(\dot{G})$.

Fix $W \prec (\mathrm{{H}}(\theta ),\in ,{\mathbb {P}},\dot{b},p)$ with $W \in \text {IA}_\kappa$. Set $\delta _W=W\cap \kappa ^+$. By the ${<}\kappa$-closure of ${\mathbb {P}}$ and Lemma 2.7, there exists a $(W,{\mathbb {P}})$-generic filter *g* containing *p*.

By the $(W,{\mathbb {P}})$-genericity of *g*, the ${<}\kappa ^+$-distributivity of ${\mathbb {P}}$, and the fact that $\dot{b} \in W$, it follows that for every $\gamma < \delta _W$, some condition $p_\gamma$ in *g* decides the value of $\dot{b} \restriction \gamma$. Define

11 $$\begin{aligned} t ~ = ~ \bigcup \{{s \in {}^{{<}\kappa ^+} \kappa ^+}~\vert ~{\exists \gamma < \delta _W ~ p_\gamma \Vdash _{\mathbb {P}}{\text {``}{\check{s} = \dot{b} \restriction \check{\gamma }}\text {''}}}\}. \end{aligned}$$

Then *t* is a function from $\delta _W$ to $\delta _W$. Moreover, by the $(W,{\mathbb {P}})$-genericity of *g* and the fact that $\dot{b}$ is forced to be a branch through $\dot{\mathcal {T}}(\dot{G}_0)$, we know that

12 $$\begin{aligned} \forall \gamma \in \delta _W \cap S^{\kappa ^+}_\kappa ~ \exists p \in g ~ p \Vdash _{\mathbb {P}}{\text {``}{\dot{b} \restriction \gamma \ne (\textstyle {\bigcup \dot{G}_0})(\gamma )}\text {''}}. \end{aligned}$$

Hence, if we let $g_0$ denote the 0-th component of the $(W,{\mathbb {P}})$-generic filter *g*, then we have $t \restriction \gamma \ne (\bigcup g_0)(\gamma )$ for all $\gamma < \delta _W$. It follows that

$$\begin{aligned} f_0 ~ = ~ (\textstyle {\bigcup g_0}) ~ \cup ~ \{(\delta _W, t)\} \end{aligned}$$

is a condition in ${\mathbb {Q}}_0$.

Let $p(f_0,g)$ be the function defined in Definition 4.15. Then $p(f_0,g)$ is not a condition in ${\mathbb {P}}$ that extends every element of *g*, because otherwise it would force that $\dot{b} \restriction \delta _W = t = (\bigcup \dot{G}_0) (\delta _W)$ and hence it would also force that $\dot{b} \restriction \delta _W \notin \dot{\mathcal {T}}(\dot{G}_0)$, contradicting our assumptions on *p*.

In this situation, Lemma 4.16 yields an $\alpha \in W \cap [1,\varepsilon )$ such that $p(f_0,g) \restriction \alpha$ is a condition in ${\mathbb {P}}_\alpha$ below every member of $g_\alpha$ and there is a condition *q* below $p(f_0,g) \restriction \alpha$ in ${\mathbb {P}}_\alpha$ with the property that whenever *G* is ${\mathbb {P}}_\alpha$-generic over $\mathrm{{V}}$ with $q\in G$, then every proper initial segment of $(\bigcup G_0)(\delta _W)$ is an element of *W*, and is an $\dot{S}_\alpha ^G$-node. Since the 0-th coordinate of *q* extends $f_0$, we know that

13 $$\begin{aligned} q \Vdash _{{\mathbb {P}}_\alpha } {\text {``}{(\textstyle {\bigcup \dot{G}_0})(\check{\delta }_W) = \check{t}}\text {''}}. \end{aligned}$$

Let $H_W$ denote the transitive collapse of *W*, and let ${\sigma }:{H_W}\longrightarrow {W \prec \mathrm{{H}}(\theta )}$ denote the inverse of the transitive collapsing map of *W*. Set $\bar{g}=\sigma ^{{-}1}[g]$, $\bar{g}_\alpha =\sigma ^{{-}1}[g_\alpha ]$, $\bar{{\mathbb {P}}}=\sigma ^{{-}1}({\mathbb {P}})$ and $\bar{{\mathbb {P}}}_\alpha =\sigma ^{{-}1}({\mathbb {P}}_\alpha )$. Let *G* be ${\mathbb {P}}_\alpha$-generic over $\mathrm{{V}}$ with $q \in G$. Since *q* extends every element of the $(W,{\mathbb {P}}_\alpha )$-generic filter $g_\alpha$, it follows that $G \cap W = g_\alpha$, $\bar{g}_\alpha$ is $\bar{{\mathbb {P}}}_\alpha$-generic over $H_W$, and the map $\sigma$ can be lifted to an elementary

$$\begin{aligned} {\hat{\sigma }}:{H_W[\bar{g}_\alpha ]}\longrightarrow {\mathrm{{H}}(\theta )[G]} \end{aligned}$$

by setting $\hat{\sigma }(\bar{\tau }^{\bar{g}_\alpha })=(\sigma (\bar{\tau }))^G$ for all $\bar{{\mathbb {P}}}_\alpha$-names $\bar{\tau }$ in $H_W$. Moreover, we know that $\bar{g}$ is $\bar{{\mathbb {P}}}$-generic over $H_W$ and the function

$$\begin{aligned} {\bar{b}= \sigma ^{{-}1}(\dot{b})^{\bar{g}}}:{\delta _W}\longrightarrow {\delta _W} \end{aligned}$$

is an element of $H_W[\bar{g}]$, but not necessarily an element of its inner model $H_W[\bar{g}_\alpha ]$.

### Claim 4.29

$$t = \bar{b}$$.

### Proof of Claim 4.29

Fix $\gamma < \delta _W$, and set $s= t \restriction \gamma$. By earlier remarks, we know that $s \in W$ and, by the definition of *t* in (11), there is $p \in g\subseteq W$ with $p \Vdash _{{\mathbb {P}}} {\text {``}{\dot{b} \restriction \check{\gamma } = \check{s}}\text {''}}$. We now know that *p*, $\dot{b}$, $\gamma$, and *s* are elements of $W = {\mathrm{{ran}}(\sigma )}$, and since ${\mathrm{{crit}}\left( {\sigma }\right) } = \delta _W$, it follows that $\sigma$ fixes $\gamma$ and *s*. The elementarity of $\sigma$ now implies that

$$\begin{aligned} \sigma ^{{-}1}(p) \Vdash _{\bar{{\mathbb {P}}}}{\text {``}{\sigma ^{{-}1}(\dot{b}) \restriction \check{\gamma } = \check{s}}\text {''}} \end{aligned}$$

holds in $H_W$. Moreover, since $p \in g$, we have $\sigma ^{{-}1}(p) \in \bar{g}$ and hence $\bar{b} \restriction \gamma = s$ holds in $H_W[\bar{g}]$. $\square$

Let $\bar{S}=\sigma ^{{-}1}(\dot{S}_\alpha )^{\bar{g}_\alpha }$. By Corollary 4 and the elementarity of ${\sigma }:{H_W}\longrightarrow {\mathrm{{H}}(\theta )}$, we know that $(S^{\delta _W}_\kappa \setminus \bar{S})^{H_W[\bar{g}_\alpha ]}$ remains stationary when going from $H_W[\bar{g}_\alpha ]$ to $H_W[\bar{g}]$. Since $\bar{b}$ maps from $\delta _W$ to $\delta _W$ and $(S^{\delta _W}_\kappa \setminus \bar{S})^{H_W[\bar{g}_\alpha ]}$ is stationary in $H_W[\bar{g}]$, there is an $\ell < \delta _W$ such that the following statements hold in $H_W[\bar{g}]$:

- $${\mathrm{{cof}}(\ell )} = \kappa $$.
- $\ell$ is closed under $\bar{b}$.
- $$\ell \notin \bar{S}$$.

Then $b_*= \bar{b} \restriction \ell$ maps from $\ell$ to $\ell$, and, by the ${<}\delta _W$-distributivity of $\bar{{\mathbb {P}}}$ over $H_W$, we know that $b_*$ is an element of $H_W$. Moreover, since ${\mathrm{{crit}}\left( {\hat{\sigma }}\right) } = \delta _W$, we have $\hat{\sigma }(b_*) = b_*$. In addition, since $\ell = {\mathrm{{dom}}(b_*)} \in (S^{\delta _W}_\kappa \setminus \bar{S})^{H_W[\bar{g}_\alpha ]}$ and $\ell$ is closed under $b^*$, we can conclude that $H_W[\bar{g}_\alpha ]$ believes that $b_*$ is not a $\bar{S}$-node.^22^ Then the elementarity of ${\hat{\sigma }}:{H_W[\bar{g}_\alpha ]}\longrightarrow {\mathrm{{H}}(\theta )[G]}$ implies that $\hat{\sigma }(b_*) = b_*$ is not a $\dot{S}_\alpha ^G$-node in $\mathrm{{H}}(\theta )[G]$. By Claim 4.29, we now have $b_* = t \restriction \ell$. So $t \restriction \ell$ is not a $\dot{S}_\alpha ^G$-node, contradicting our earlier arguments. $\square$

We are now ready to complete the proof of the main technical result of this paper.

### Proof of Theorem 4.2

Let $\kappa$ be an infinite regular cardinal and let ${\mathbb {P}}={\mathbb {P}}_\varepsilon$ be the poset constructed in Definition 4.12. Then Lemma 4.20 shows that part (1) of the theorem holds.

Next, we prove part (2) of the theorem. We will prove that the poset ${\mathbb {P}}$ is $(2^\kappa )^+$-*stationarily layered* (see [3,  Definition 29]) in $\mathrm{{V}}$, which, by [3,  Lemma 4], implies that ${\mathbb {P}}$ is $(2^\kappa )^+$-Knaster. A poset ${\mathbb {R}}$ is $\lambda$-stationarily layered if for some sufficiently large regular cardinal $\theta$, there are stationarily-many $M \in {\wp }^*_{\lambda }(\mathrm{{H}}(\theta ))$ such that $M \cap {\mathbb {R}}$ is a regular suborder of ${\mathbb {R}}$. Equivalently, we can demand that every condition *p* in ${\mathbb {R}}$ has a *reduction* into $M \cap {\mathbb {R}}$, i.e. there exists $q \in M \cap {\mathbb {R}}$ such that all extensions of *q* in $M \cap {\mathbb {R}}$ are compatible with *p* in ${\mathbb {P}}$.

For all sufficiently large regular cardinals $\theta$, the set

$$\begin{aligned} R ~ = ~ \{{M \in {\wp }^*_{(2^\kappa )^+}(\mathrm{{H}}(\theta ))}~\vert ~{M\prec \mathrm{{H}}(\theta ), ~{}^{\kappa } M \subseteq M, ~ {\mathbb {P}}\in M}\} \end{aligned}$$

is stationary in ${\wp }_{(2^\kappa )^+}(\mathrm{{H}}(\theta ))$.

We prove that *R* witnesses the $(2^\kappa )^+$-stationary layeredness of ${\mathbb {P}}$. Fix $M \in R$ and a condition *p* in ${\mathbb {P}}$. By the density of flat conditions in ${\mathbb {P}}$, we we may assume that there exists a sequence $\langle {x_\alpha }~\vert ~{\alpha \in {\mathrm{{sprt}}(p)}}\rangle$ that witnesses that *p* is flat.

Furthermore, we may assume thatholds for all $\alpha \in {\mathrm{{sprt}}(p)}$, because redefining *p* in this way results in a condition equivalent to *p*.

Set $s= M \cap {\mathrm{{sprt}}(p)}$ and define $q= p \restriction s$.

### Claim 4.30

The condition *q* is a reduction of *p* into $M\cap {\mathbb {P}}$.

### Proof of Claim 4.30

First, we verify that *q* is an element of *M*. Since we have $x_\alpha \in \mathrm{{H}}(\kappa ^+) \subseteq M$ for all $\alpha \in {\mathrm{{sprt}}(p)}$, the closure properties of *M* imply that the sequence $\langle {x_\alpha }~\vert ~{\alpha \in s}\rangle$ is an element of *M*. Since the condition *q* is definable from the sequence $\langle {x_\alpha }~\vert ~{\alpha \in s}\rangle$, it follows that *q* is also an element of *M*.

Next, assume *r* is a condition in $M\cap {\mathbb {P}}$ below *q*. Let $p \wedge r$ denote the natural amalgamation of *p* and *r*, i.e. we have $(p \wedge r)(\beta )= r(\beta )$ for all $\beta \in {\mathrm{{sprt}}(r)}$, and $(p \wedge r)(j) = p(\beta )$ for all $\beta \in {\mathrm{{sprt}}(p)} \setminus {\mathrm{{sprt}}(r)}$. Since $p \wedge r$ is clearly a function whose support has size at most $\kappa$, it is a condition in ${\mathbb {P}}$. We verify that $p \wedge r$ is below both *p* and *r* in ${\mathbb {P}}$ by checking inductively that $(p \wedge r) \restriction \beta$ is below both $p \restriction \beta$ and $p \restriction \beta$ for all $\beta \le \varepsilon$. Suppose this statement holds at all $\alpha < \beta \le \varepsilon$. Clearly, if $\beta$ is a limit ordinal, then it holds at $\beta$ as well. Hence, we may assume that $\beta =\alpha +1$ and that $(p \wedge r) \restriction \alpha$ lies below both $p \restriction \alpha$ and $r \restriction \alpha$. If $\alpha$ is not in the support of either *p* or *r*, then the above statement trivially holds at $\beta$ as well. Hence, we have to consider the following two cases:

**Case 1:** $\alpha \in {\mathrm{{sprt}}(r)}$. By definition of the condition $p \wedge r$, we have $(p \wedge r)(\alpha ) = r(\alpha )$ and $r \le _{\mathbb {P}}q$ implies that $r \restriction \alpha \Vdash _{{\mathbb {P}}_\alpha }{\text {``}{r(\alpha )\le _{\dot{{\mathbb {Q}}}_\alpha }q(\alpha )}\text {''}}$. Since $(p \wedge r) \restriction \alpha \le _{{\mathbb {P}}_\alpha } r \restriction \alpha$ by our induction hypothesis, this shows that

14 $$\begin{aligned} (p \wedge r) \restriction \alpha \Vdash _{{\mathbb {P}}_\alpha }{\text {``}{(p \wedge r)(\alpha ) \le _{\dot{{\mathbb {Q}}}_\alpha } q(\alpha )}\text {''}}. \end{aligned}$$

Moreover, since $r \in M$ and $\vert {{\mathrm{{sprt}}(r)}}\vert \le \kappa \subseteq M$, we have $\alpha \in {\mathrm{{sprt}}(r)} \subseteq M$. In particular, if $\alpha \in {\mathrm{{sprt}}(p)}$, then $\alpha \in s$ and hence $p(\alpha ) = q(\alpha )$. In combination with (14), this yields

15 $$\begin{aligned} (p \wedge r) \restriction \alpha \Vdash _{{\mathbb {P}}_\alpha }{\text {``}{(p \wedge r)(\alpha ) \le _{\dot{{\mathbb {Q}}}_\alpha } p(\alpha )}\text {''}}. \end{aligned}$$

In the other case, if $\alpha \notin {\mathrm{{sprt}}(p)}$, then (15) holds trivially.

**Case 2:** $\alpha \in {\mathrm{{sprt}}(p)}\setminus {\mathrm{{sprt}}(r)}$. By the definition of $(p \wedge r)$, we have $(p \wedge r)(\alpha ) = p(\alpha )$. Since $\alpha \notin \text {sprt}(r)$, it follows trivially that

$$\begin{aligned} (p\wedge r)\restriction \alpha \Vdash _{{\mathbb {P}}_\alpha }{\text {``}{(p\wedge r)(\alpha )\le _{\dot{{\mathbb {Q}}}_\alpha }p(\alpha )\le _{\dot{{\mathbb {Q}}}_\alpha }r(\alpha )}\text {''}}. \end{aligned}$$

These computations show that *q* is a reduction of *p* into $M \cap {\mathbb {P}}$. $\square$

This concludes the proof that the poset is ${\mathbb {P}}$ is $(2^\kappa )^+$-stationarily layered, and hence $(2^\kappa )^+$-Knaster.

We now verify part (3a) of the theorem. Let *G* be ${\mathbb {P}}$-generic over $\mathrm{{V}}$. Suppose *S* is a bistationary subset of $S^{\kappa ^+}_\kappa$ in $\mathrm{{V}}[G]$ such that $S^{\kappa ^+}_\kappa \setminus S$ contains a stationary set *T* in $I[\kappa ^+]$ in $\mathrm{{V}}[G]$. Pick a club *D* in $\kappa ^+$ and a $\kappa ^+$-sequence $\vec {z}$ of sets from $[\kappa ^+]^{{<}\kappa }$ witnessing that *T* is an element of $I[\kappa ^+]$ in $\mathrm{{V}}[G]$. A combination of Lemma 4.20 and part (2) of this theorem now shows that there is a subset $P\in \mathrm{{V}}$ of $S^{\kappa ^+}_\kappa \times 2^\kappa$ and a sequence $\langle {q_{\gamma ,\xi }}~\vert ~{(\gamma ,\xi )\in P}\rangle \in \mathrm{{V}}$ of flat conditions in ${\mathbb {P}}$ such that $\dot{B}^G=S$, where $\dot{B}$ is the ${\mathbb {P}}$-name $\{{(\check{\gamma },q_{\gamma ,\xi })}~\vert ~{(\gamma ,\xi )\in P}\}$. Pick an element *s* of the set $\mathcal {N}$ defined before Definition 4.12 such that ${\mathrm{{dom}}(s)}=P$ and, if $(\gamma ,\xi )\in P$, then ${\mathrm{{dom}}(s(\gamma ,\xi ))}={\mathrm{{sprt}}(q_{\gamma ,\xi })}$ and $q_{\gamma ,\xi }(\ell )=\check{x}$ for all $\ell \in {\mathrm{{dom}}(s(\gamma ,\xi ))}$ with $b(\gamma ,\xi )(\ell )=x$. By our assumptions on $\varepsilon$ and *b*, part (2) of this theorem allows us to find $0<\alpha <\varepsilon$ with the property that $\dot{B}$ is a ${\mathbb {P}}_\alpha$-name, $b(\alpha )=s$ and $\vec {z},D,T\in \mathrm{{V}}[G_\alpha ]$, where $G_\alpha$ is the filter on ${\mathbb {P}}_\alpha$ induced by *G*. Clearly, the fact that *T* is bistationary in $\mathrm{{V}}[G]$ implies that *T* is bistationary in $\mathrm{{V}}[G_\alpha ]$. Moreover, since $\mathrm{{V}}[G]$ and $\mathrm{{V}}[G_\alpha ]$ have the same $\kappa$-sequences of ordinals, every element of $D \cap T$ is also approachable with respect to $\vec {z}$ in $\mathrm{{V}}[G_\alpha ]$. Hence, we know that $S^{\kappa ^+}_\kappa \setminus S$ contains a stationary set in $I[\kappa ^+]$ in $\mathrm{{V}}[G_\alpha ]$. Since our choice of $\alpha$ ensures that $\dot{B}_\alpha =\dot{B}$, we can conclude that $\dot{S}_\alpha ^{G_\alpha }=\dot{B}_\alpha ^{G_\alpha }=\dot{B}^G=S$ and hence forcing with $\dot{{\mathbb {Q}}}_\alpha ^{G_\alpha }$ over $\mathrm{{V}}[G_\alpha ]$ adds an order-preserving function from *T*(*S*) to $\mathcal {T}(G_0)$.

Finally, we prove part (3b) of the theorem. Hence, assume that $\kappa ^{{<}\kappa } \le \kappa ^+$ holds in $\mathrm{{V}}$ and fix an enumeration $\vec {z} = \langle {z_\xi }~\vert ~{\xi < \kappa ^+}\rangle$ of all elements of $[\kappa ^+]^{{<}\kappa }$ in $\mathrm{{V}}$. By Lemma 2.10, the set *M* of all $\gamma \in S^{\kappa ^+}_\kappa$ that are approachable with respect to $\vec {z}$ is a maximal element of $I[\kappa ^+] \cap {\wp }({S^{\kappa ^+}_\kappa })$ mod ${{NS}}_{}$ in $\mathrm{{V}}$. Since ${\mathbb {P}}$ is ${<}\kappa ^+$-distributive and therefore $\vec {z}$ still enumerates all of $[\kappa ^+]^{<\kappa }$ in $\mathrm{{V}}[G]$, it follows that *M* is still the set of all $\gamma \in S^{\kappa ^+}_\kappa$ that are approachable with respect to $\vec {z}$ in *V*[*G*], and hence *M* is still a maximal element of $I[\kappa ^+] \cap {\wp }({S^{\kappa ^+}_\kappa })$ mod ${{NS}}_{}$ in $\mathrm{{V}}[G]$.

Now, suppose that $S \in \mathrm{{V}}[G]$ is bistationary in $S^{\kappa ^+}_\kappa$ and $M \setminus S$ is stationary. Since $M \in I[\kappa ^+]$ and $I[\kappa ^+]$ is an ideal, it follows that $M \setminus S \in I[\kappa ^+]$. So $M \setminus S$ is a stationary set in $I[\kappa ^+]$. Hence by part (3a) of the theorem, there is an order-preserving function from *T*(*S*) to $\mathcal {T}(G_0)$ in *V*[*G*]. $\square$

## Applications

We now apply Theorem 4.2 to prove the results presented in the introduction of the paper.

### Corollary 5

Let $\kappa$ be an infinite regular cardinal satisfying $\kappa ^{{<}\kappa } \le \kappa ^+$, let ${\mathbb {P}}$ be the partial order given by Theorem 4.2 and let *M* be a maximum element of $I[\kappa ^+] \cap {\wp }({S^{\kappa ^+}_\kappa })$ mod ${{NS}}_{}$. If *G* is ${\mathbb {P}}$-generic over $\mathrm{{V}}$, then the set ${{NS}}_{}\restriction M$ is $\varvec{\Delta }_1(\mathrm{{H}}((2^\kappa )^+))$-definable in $\mathrm{{V}}[G]$.

### Proof

Work in $\mathrm{{V}}[G]$ and let *T* be the subtree of ${}^{{<}\kappa ^+}\kappa ^+$ given by Theorem 4.2. Then $T\subseteq {}^{{<}\kappa ^+}\kappa ^+\in \mathrm{{H}}((2^\kappa )^+)$. Define $\mathcal {S}$ to be the collection of all subsets *A* of *M* such that either there exists a closed unbounded subset *C* of $\kappa ^+$ with $C\cap M\subseteq A$ or there exists an order-preserving function from the tree $T(S^{\kappa ^+}_\kappa \setminus A)$ into the tree *T*.

Then the set $\mathcal {S}$ is definable by a $\Sigma _1$-formula with parameters *M*, *T* and ${}^{{<}\kappa ^+}\kappa ^+$.

### Claim 5.1

The set $\mathcal {S}$ is equal to the collection of all subsets of *M* that are stationary in $\kappa ^+$.

### Proof of Claim 5.1

First, let $A\subseteq M$ be stationary in $\kappa ^+$ with the property that there is no club *C* in $\kappa ^+$ with $C\cap M\subseteq A$. Since *M* is stationary in $\kappa ^+$, this shows that *A* is bistationary in $S^{\kappa ^+}_\kappa$, $M\setminus A$ is stationary, and hence Theorem 4.2 yields an order-preserving function from $T(S^{\kappa ^+}_\kappa \setminus A)$ into *T* that witnesses that *A* is contained in $\mathcal {S}$. This argument shows that $\mathcal {S}$ contains all stationary subsets of *M*.

Now, assume, towards a contradiction, that there is a non-stationary subset *A* of $\kappa ^+$ that is contained in $\mathcal {S}$. Then there is an order-preserving embedding of $T(S^{\kappa ^+}_\kappa \setminus A)$ into *T* and a closed unbounded subset *C* of $\kappa ^+$ with $A\cap C=\emptyset$. But then $C\cap S^{\kappa ^+}_\kappa$ is a $\kappa$-club that is a subset of $S^{\kappa ^+}_\kappa \setminus A$ and, by earlier remarks, the tree $T(S^{\kappa ^+}_\kappa \setminus A)$ contains a cofinal branch. But then the tree *T* also contains a cofinal branch, a contradiction. $\square$

By the above claim, the set ${{NS}}_{}\restriction M={\wp }({M})\setminus \mathcal {S}$ is definable by a $\Pi _1$-formula with parameters in $\mathrm{{H}}((2^\kappa )^+)$. $\square$

In particular, the above corollary directly shows how the definability results of [13] and [23] can be derived from Theorem 4.2.

### Corollary 6

Let $\kappa$ be an infinite regular cardinal satisfying $\kappa ^{{<}\kappa } = \kappa$ and let ${\mathbb {P}}$ be the poset given by Theorem 4.2. If *G* is ${\mathbb {P}}$-generic over $\mathrm{{V}}$, then ${{NS}}_{} \restriction S^{\kappa ^+}_\kappa$ is $\varvec{\Delta }_1(\mathrm{{H}}((2^\kappa )^+))$-definable in $\mathrm{{V}}[G]$.

### Proof

By Lemma 2.10, if $\kappa ^{{<}\kappa } = \kappa$ holds in $\mathrm{{V}}$, then $S^{\kappa ^+}_\kappa$ is a maximum element of $I[\kappa ^+] \cap {\wp }({S^{\kappa ^+}_\kappa })$ mod ${{NS}}_{}$. Since forcing with ${\mathbb {P}}$ does not change cofinalities below $\kappa ^+$, the desired conclusion directly follows from Corollary 5. $\square$

The following lemma establishes a connection between principles of stationary reflection and the $\Pi _1$-definability of restrictions of the non-stationary ideals that will be crucial for proofs of our main results.

### Lemma 5.2

Let *S* be a stationary subset of an uncountable regular cardinal $\delta$ and let $\mathcal {E}$ be a set of stationary subsets of $S^\delta _{{>}\omega }$ with the property that for every stationary subset *A* of *S*, there exists $E\in \mathcal {E}$ such that *A* reflects at every element of *E*.

If $\mathcal {E}$ is definable by a $\Sigma _1$-formula with parameter *p*, then the set ${{NS}}_{}\restriction S$ is definable by a $\Pi _1$-formula with parameters *p*, *S* and $\mathrm{{H}}(\delta )$.

### Proof

Let $\mathcal {S}$ denote the collection of all subsets *A* of *S* with the property that there exists $E\in \mathcal {E}$ such that $A\cap \alpha$ is stationary in $\alpha$ for all $\alpha \in E$. By our assumptions on $\mathcal {E}$, the set $\mathcal {S}$ is definable by a $\Sigma _1$-formula with parameters *p*, *S* and $\mathrm{{H}}(\delta )$.

If $A\subseteq S$ is stationary in $\delta$, then our assumptions on $\mathcal {E}$ ensure that *A* is contained in $\mathcal {S}$.

In the other direction, if $E\in \mathcal {E}$ witnesses that *A* is an element of $\mathcal {S}$ and *C* is closed unbounded in $\delta$, then there is $\alpha \in E\cap \mathrm{{Lim}}(C)$ with $A\cap \alpha$ stationary in $\alpha$ and hence $\emptyset \ne A\cap C\cap \alpha \subseteq A\cap C$. Together, this shows that $\mathcal {S}$ is equal to the collection of all subsets of *S* that are stationary in $\delta$ and hence ${{NS}}_{}\restriction S={\wp }({\delta })\setminus \mathcal {S}$ is definable by a $\Pi _1$-formula with parameters *p*, *S* and $\mathrm{{H}}(\delta )$. $\square$

The above lemma directly shows that strong forms of stationary reflection cause restrictions of non-stationary ideals to be $\varvec{\Delta }_1$-definable.

### Corollary 7

Let $\delta$ be an uncountable regular cardinal, let *E* be a stationary subset of $S^\delta _{{>}\omega }$ and let *S* be a stationary subset of $\delta$ such that every stationary subset of *S* reflects almost everywhere in *E* (i.e. for every stationary subset *A* of *S*, there is a closed unbounded subset *C* of $\delta$ with the property that *A* reflects at every element of $C\cap E$). Then the set ${{NS}}_{}\restriction S$ is definable by a $\Pi _1$-formula with parameters *E*, *S* and $\mathrm{{H}}(\delta )$.

### Proof

If we define $\mathcal {E}=\{{C\cap E}~\vert ~{C \,{ club\, in }\, \delta }\}$, then $\mathcal {E}$ is definable by a $\Sigma _1$-formula with parameter *E* and this shows that the sets $\mathcal {E}$ and *S* satisfy the assumptions of Lemma 5.2. $\square$

Note that a classical result of Magidor in [22] shows that, starting with a weakly compact cardinal, it is possible to construct a model of set theory in which every stationary subset of $S^2_0$ reflects almost everywhere in $S^2_1$. The above corollary shows that the set ${{NS}}_{}\restriction S^2_0$ is $\varvec{\Delta }_1(\mathrm{{H}}(\omega _3))$-definable in Magidor’s model.

The next theorem will be used to derive Theorem 1.2.

### Theorem 5.3

Assume that $2^{\omega _1}=\omega _2$, and $\theta$ is a cardinal with $\theta ^{\omega _2}=\theta$.

Then there exists a ${<}\omega _2$-directed closed, cardinal-preserving poset ${\mathbb {P}}$ with the property that the following statements hold in $\mathrm{{V}}[G]$ whenever *G* is ${\mathbb {P}}$-generic over $\mathrm{{V}}$:

1. $$2^{\omega _2}=\theta $$.
2. If for every stationary subset *A* of $S^2_0$, there is a stationary subset *R* of $\text {IA}_{\omega _1}$ such that $W \prec H(\omega _3)$ and *A* reflects at $W \cap \omega _2$ for all $W \in R$, then the set ${{NS}}_{}\restriction S^2_0$ is $\varvec{\Delta }_1(\mathrm{{H}}(\omega _3))$-definable.

### Proof

Let *G* be $\mathrm{{Add}}({\omega _2},{\theta })$-generic over $\mathrm{{V}}$. Since $2^{\omega _1}=\omega _2$ holds in $\mathrm{{V}}$, we know that $\mathrm{{Add}}({\omega _2},{\theta })$ satisfies the $\omega _3$-chain condition in $\mathrm{{V}}$ and hence all cofinalities are preserved in $\mathrm{{V}}[G]$. Work in $\mathrm{{V}}[G]$. Then our assumptions ensure that $2^{\omega _1}=\omega _2$ and $2^{\omega _2}=\theta =\theta ^{\omega _2}$. Let ${\mathbb {P}}$ be the poset given by Theorem 4.2 for $\kappa =\omega _1$ and $\varepsilon =\theta$, and let *M* be a maximum element of $I[\omega _2] \cap {\wp }({S^{\omega _2}_{\omega _1}})$ mod ${{NS}}_{}$, which exists due to the assumption that $2^\omega \le \omega _2$ (see Lemma 2.10). Then Lemma 3.6 and part (1) of Theorem 4.2 show that ${\mathbb {P}}$ is forcing equivalent to a ${<}\omega _2$-directed closed poset. Moreover, since $2^{\omega _1}=\omega _2$ holds, part (2) of Theorem 4.2 shows that ${\mathbb {P}}$ satisfies the $\omega _3$-chain condition. Finally, Lemma 4.20 shows that ${\mathbb {P}}$ has a dense subset of cardinality $\theta$.

Now, let *H* be ${\mathbb {P}}$-generic over $\mathrm{{V}}[G]$ and work in $\mathrm{{V}}[G,H]$. By the above observations, we then have $2^{\omega _2}=\theta$. In addition, part (3b) of Theorem 4.2 shows that *M* is the maximum element of $I[\omega _2] \cap {\wp }({S^{\omega _2}_{\omega _1}})$ mod ${{NS}}_{}$. Moreover, Corollary 5 shows that ${{NS}}_{}\restriction M$ is $\varvec{\Delta }_1(\mathrm{{H}}(\omega _3))$-definable. In the following, assume that for every stationary subset *A* of $S^2_0$, there is a stationary subset *R* of $\text {IA}_{\omega _1}$ such that $W \prec H(\omega _3)$ and *A* reflects at $W \cap \omega _2$ for all $W \in R$. Set $\mathcal {E}={\wp }({M})\setminus {{NS}}_{\omega _2}$. Then $\mathcal {E}$ is definable by a $\Sigma _1$-formula with parameters in $\mathrm{{H}}(\omega _3)$.

### Claim 5.4

For every stationary subset *A* of $S^2_0$, there is an element *E* of $\mathcal {E}$ with the property that *A* reflects at every element of *E*.

### Proof of the Claim

By our assumption, there is a stationary subset *R* of $\text {IA}_{\omega _1}$ such that $W \prec H(\omega _3)$ and *A* reflects at $W \cap \omega _2$ for every $W \in R$. If we now define $E_0=\{{W \cap \omega _2}~\vert ~{W \in R}\}$, then $E_0$ is a stationary subset of $S^{\omega _2}_{\omega _1}$. Moreover, since $2^\omega \le \omega _2$, each $W \in R$ has (as an element) an enumeration $\vec {z} = \langle {z_\xi }~\vert ~{\xi <\omega _2}\rangle$ of $[\omega _2]^\omega$ and therefore the internal approachability of *W* and the fact that $\vec {z} \in W$ imply that $W \cap \omega _2$ is approachable with respect to $\vec {z}$. Hence, the set $E_0$ is stationary and an element of $I[\omega _2]$. Since *M* is the largest such element mod ${{NS}}_{}$, we have in particular that $E= E_0 \cap M$ is a stationary subset of *M*. $\square$

Using Lemma 5.2, we can now conclude that ${{NS}}_{}\restriction S^2_0$ is definable by a $\Sigma _1$-formula with parameters in $\mathrm{{H}}(\omega _3)$. $\square$

### Proof of Theorem 1.2

Assume that $\mathrm{{FA}}$ holds, where $\mathrm{{FA}}$ is one of the following axioms:

- $\mathrm{{MM}}^{+\mu }$, where $\mu$ is a cardinal and $0 \le \mu \le \omega _1$; or
- $\mathrm{{PFA}}^{+\mu }$, where $\mu$ is a cardinal and $1 \le \mu \le \omega _1$.

Let $\theta$ be a cardinal with $\theta ^{\omega _2}=\theta$. Since $\mathrm{{PFA}}$ implies that $2^\omega =2^{\omega _1}=\omega _2$ holds (see [15,  Theorem 16.20 & 31.23]), our assumption allows us to apply Theorem 5.3 to obtain a ${<}\omega _2$-directed closed poset with the properties listed in the conclusion of theorem. Let *G* be ${\mathbb {P}}$-generic over $\mathrm{{V}}$. Then [4,  Theorem 4.7] ensures that $\mathrm{{FA}}$ holds in $\mathrm{{V}}[G]$. Since $\mathrm{{FA}}$ holds in $\mathrm{{V}}[G]$, there exists a stationary subset *R* of $\text {IA}_{\omega _1}$ with the property that for all $W \in R$, we have $W \prec H(\omega _3)$ and *A* reflects at $W \cap \omega _2$.^23^ Then Theorem 5.3 allows us to conclude that ${{NS}}_{}\restriction S^2_0$ is $\varvec{\Delta }_1(\mathrm{{H}}(\omega _3))$-definable in $\mathrm{{V}}[G]$. $\square$

The next theorem will be used to derive Theorem 1.3.

### Theorem 5.5

Assume that $2^\omega =\omega _1$, $2^{\omega _1}=\omega _2$, and $\theta$ is a cardinal with $\theta ^{\omega _2}=\theta$.

Then there exists a ${<}\omega _2$-directed closed, cardinal-preserving poset ${\mathbb {P}}$ with the property that the following statements hold in $\mathrm{{V}}[G]$ whenever *G* is ${\mathbb {P}}$-generic over $\mathrm{{V}}$:

1. $$2^{\omega _2}=\theta $$.
2. If every stationary subset of $S^2_0$ reflects to a point in $S^2_1$, then the set ${{NS}}_{\omega _2}$ is $\varvec{\Delta }_1(\mathrm{{H}}(\omega _3))$-definable.

### Proof

Let *G* be $\mathrm{{Add}}({\omega _2},{\theta })$-generic over $\mathrm{{V}}$, let ${\mathbb {P}}$ be the poset produced by an application of Theorem 4.2 with $\kappa =\omega _1$ and $\varepsilon =\theta$ in $\mathrm{{V}}[G]$, and let *H* be ${\mathbb {P}}$-generic over $\mathrm{{V}}[G]$. As above, we have $(2^{\omega _2})^{\mathrm{{V}}[G,H]}=\theta$ and, since $2^\omega =\omega _1$ holds in $\mathrm{{V}}[G]$, part (4) of Lemma 2.10 and part (3b) of Theorem 4.2 imply that $(S^2_1)^{\mathrm{{V}}[G]}=(S^2_1)^{\mathrm{{V}}[G,H]}$ is a maximum element of $I[\omega _2] \cap {\wp }({S^2_1})$ mod ${{NS}}_{}$ in both $\mathrm{{V}}[G]$ and $\mathrm{{V}}[G,H]$. In particular, Corollary 5 implies that ${{NS}}_{}\restriction S^2_1$ is $\varvec{\Delta }_1(\mathrm{{H}}(\omega _3))$-definable in $\mathrm{{V}}[G,H]$.

Now, work in $\mathrm{{V}}[G,H]$ and assume that every stationary subset of $S^2_0$ reflects to a point in $S^2_1$. Then every stationary subset of $S^2_0$ reflects to stationary-many points in $S^2_1$ and we can apply Lemma 5.2 with $S^2_0$ and ${\wp }({S^2_1})\setminus {{NS}}_{\omega _2}$ to show that ${{NS}}_{}\restriction S^2_0$ is $\varvec{\Delta }_1(\mathrm{{H}}(\omega _3))$-definable. Since it is easy to see that

$$\begin{aligned} {{NS}}_{\omega _2} ~ = ~ \{{A\subseteq \omega _2}~\vert ~{A\cap S^2_0\in {{NS}}_{}\restriction S^2_0 \text { and } A\cap S^2_1\in {{NS}}_{}\restriction S^2_1}\}, \end{aligned}$$

these computations allow us to conclude that ${{NS}}_{\omega _2}$ is $\varvec{\Delta }_1$-definable. $\square$

### Proof of Theorem 1.3

Assume that $2^\omega =\omega _1$, $2^{\omega _1}=\omega _2$ and either $\mathrm{{FA}}^{{+}}(\sigma \text {-closed})$ or $\mathrm {SCFA}$ holds. Let $\theta$ be a cardinal with $\theta ^{\omega _2}=\theta$, let ${\mathbb {P}}$ be the poset produced by an application of Theorem 5.5 and let *G* be ${\mathbb {P}}$-generic over $\mathrm{{V}}$. Then $(2^{\omega _2})^{\mathrm{{V}}[G]}=\theta$ holds. Moreover, by Theorem 4.7 of [4], either $\mathrm{{FA}}^{{+}}(\sigma \text {-closed})$ or $\mathrm {SCFA}$ holds in $\mathrm{{V}}[G]$. In the case of $\mathrm {SCFA}$, the fact that CH also holds ensures (by [11,  Theorem 2.7 and Observation 2.8]) that, in $\mathrm{{V}}[G]$, every stationary subset of $S^2_0$ reflects in a point in $S^2_1$.^24^ In the case where $\text {FA}^+\big ( \sigma \text {-closed} \big )$ holds, the proof of Theorem 8.3 of [2] ensures the same kind of stationary reflection.

By Theorem 5.5, this shows that ${{NS}}_{\omega _2}$ is $\varvec{\Delta }_1$-definable in $\mathrm{{V}}[G]$. $\square$

## Footnotes

1 In keeping with the prevailing convention in the literature: $\text {MM}^+$ refers to $\text {MM}^{+1}$, but $\text {MM}^{++}$ refers to $\text {MM}^{+\omega _1}$, *not* to $\text {MM}^{+2}$ (and similarly for PFA and other forcing axioms).

2 Sometimes the requirement that $g \subseteq W$ is dropped, but then one has the demand that $D\cap g \cap W\ne \emptyset$ holds for each dense $D \in W$.

3 Note that this could fail if *W* were allowed to have non-transitive intersection with $\tau$.

4 We do not require here that $p_{\alpha +1}$ is a total master condition for $N_\alpha$. That is, if $D \in N_\alpha$ is dense, the upward closure of $p_{\alpha +1}$ is only required to meet *D*, not necessarily $D \cap N_\alpha$.

5 Note that such an enumeration exists by our cardinal arithmetic assumption.

6 E.g. if $2^{\omega _1} = \omega _2$ then there is a natural $\text {IA}_{\omega _1}$-proper poset that forces the *Approachability Property* to hold at $\omega _2$, hence destroys the Proper Forcing Axiom. This poset is just the natural poset to shoot an $\omega _1$-club through the set *M* described in Lemma 2.10.

7 Jensen’s notes say this is equivalent to a definition of Shelah in [25,  Chapter 10].

8 Note that $\sigma$-closure is equivalent to $\sigma$-directed closure, so the distinction is only important at larger cardinals.

9 Note that this clause is allowed to be **vacuously** true for some elements *W* of ${\wp }^*_{\tau }(\mathrm{{H}}(\theta ))$, even for stationarily-many such sets *W*.

10 In particular, Lemma 3.6 shows that the poset ${\mathbb {P}}$ is forcing equivalent to a ${<}\kappa ^+$-directed closed poset.

11 Such a subset *M* exists by Lemma 2.10.

12 Since ${f(\delta )}:{\delta }\longrightarrow {\delta }$ and ${f(\eta )}:{\eta }\longrightarrow {\eta }$, this just means that $f(\delta )$ and $f(\eta )$ disagree at some $\xi < \delta$.

13 I.e. a model of $\mathrm{{ZFC}}$ in which $\mathrm{{V}}[G]$ is a transitive class.

14 The domain of $X(\alpha )$ is required to be a successor ordinal, so $X(\alpha )$ cannot be the entire function $(\bigcup G_0)(\alpha )$.

15 This was the only place in the argument where requirement (3(a)iii) from Definition 4.8 played a role. This requirement was used to fix the error from [23]. It ensures that the function $h_K$ is total.

16 This notation is chosen to keep in line with the notational convention from [13] of identifying ${\mathbb {P}}_1$ with ${\mathbb {Q}}_0$ and referring to the induced ${\mathbb {Q}}_0$-generic filter by $G_0$. Notice that $\bigcup g_0$ is easily a condition in ${\mathbb {Q}}_0$.

17 The idea behind this definition is that $\dot{c}_{g,\alpha }$ names the evaluation of the $\alpha$-*th component* of *g* after forcing with ${\mathbb {P}}_\alpha$ over $\mathrm{{V}}$, and $\dot{h}_{g,\alpha }$ and $\dot{X}_{g,\alpha }$ name the unions of the left and right components (respectively) of that $\alpha$-th component of *g*.

18 E.g. if $2^{\omega _1} = \omega _2$, one can code $\text {IA}_{\omega _1} \cap {\wp }_{\omega _2}(\mathrm{{H}}(\omega _2))$ as a stationary subset *S* of $S^2_1$. Then, shooting an $\omega _1$-club through *S* with initial segments is $\text {IA}_{\omega _1}$-totally proper, but kills $\mathrm{{PFA}}$.

19 Recall Remark 4.9 showing that, for successor ordinals, the right coordinate of a condition does not have to agree with $G_0$).

20 Such a lower bound exists by Lemma 4.26.

21 The length of this initial segment of *t* might depend on the given initial segment of $\vec {p}$.

22 Recall from Definition 4.8 that a function *s* is an *S*-node if no element of $S^{\kappa ^+}_\kappa \setminus S$ is closed under *s*

23 For the case corresponding to $\mathrm{{MM}}$, this follows by the proof of [6,  Theorem 13]. For the case corresponding to $\mathrm{{PFA}}^{+\mu }$ where $\mu \ge 1$, it follows from the remark on [6, p. 20]. The $\omega _1$-enumerations in both proofs are easily seen to be internally approachable enumerations.

24 Note that the $\mathrm{{CH}}$ assumption seems to be required for this consequence of SCFA; see Fuchs [12] for some corrections on previous literature.
